# The LEA gene family in tomato and its wild relatives: genome-wide identification, structural characterization, expression profiling, and role of *SlLEA6* in drought stress

**DOI:** 10.1186/s12870-022-03953-7

**Published:** 2022-12-19

**Authors:** Chunping Jia, Bin Guo, Baike Wang, Xin Li, Tao Yang, Ning Li, Juan Wang, Qinghui Yu

**Affiliations:** 1 Institute of Horticulture Crops, Xinjiang Academy of Agricultural Sciences (Key Laboratory of Genome Research and Genetic Improvement of Xinjiang Characteristic Fruits and Vegetables), Urumqi, China; 2 College of Life Science and Technology, Xinjiang University, Urumqi, China; 3 College of Computer and Information Engineering, Xinjiang Agricultural University, Urumqi, China

**Keywords:** Cultivated tomato, Wild tomato, LEA, Genome-wide identification, Expression patterns, Evolutionary analyses, Abiotic stressors, Phytohormone treatments

## Abstract

**Background:**

Late embryogenesis abundant (LEA) proteins are widely distributed in higher plants and play crucial roles in regulating plant growth and development processes and resisting abiotic stress. Cultivated tomato (*Solanum lycopersicum*) is an important vegetable crop worldwide; however, its growth, development, yield, and quality are currently severely constrained by abiotic stressors. In contrast, wild tomato species are more tolerant to abiotic stress and can grow normally in extreme environments. The main objective of this study was to identify, characterize, and perform gene expression analysis of LEA protein families from cultivated and wild tomato species to mine candidate genes and determine their potential role in abiotic stress tolerance in tomatoes.

**Results:**

Total 60, 69, 65, and 60 *LEA* genes were identified in *S. lycopersicum*, *Solanum pimpinellifolium*, *Solanum pennellii*, and *Solanum lycopersicoides*, respectively. Characterization results showed that these genes could be divided into eight clusters, with the LEA_2 cluster having the most members. Most *LEA* genes had few introns and were non-randomly distributed on chromosomes; the promoter regions contained numerous *cis*-acting regulatory elements related to abiotic stress tolerance and phytohormone responses. Evolutionary analysis showed that *LEA* genes were highly conserved and that the segmental duplication event played an important role in evolution of the *LEA* gene family. Transcription and expression pattern analyses revealed different regulatory patterns of *LEA* genes between cultivated and wild tomato species under normal conditions. Certain *S. lycopersicum LEA* (*SlLEA*) genes showed similar expression patterns and played specific roles under different abiotic stress and phytohormone treatments. Gene ontology and protein interaction analyses showed that most *LEA* genes acted in response to abiotic stimuli and water deficit. Five SlLEA proteins were found to interact with 11 *S. lycopersicum* WRKY proteins involved in development or resistance to stress. Virus-induced gene silencing of *SlLEA6* affected the antioxidant and reactive oxygen species defense systems, increased the degree of cellular damage, and reduced drought resistance in *S. lycopersicum*.

**Conclusion:**

These findings provide comprehensive information on LEA proteins in cultivated and wild tomato species and their possible functions under different abiotic and phytohormone stresses. The study systematically broadens our current understanding of LEA proteins and candidate genes and provides a theoretical basis for future functional studies aimed at improving stress resistance in tomato.

**Supplementary Information:**

The online version contains supplementary material available at 10.1186/s12870-022-03953-7.

## Background

Abiotic stressors such as drought, salinity, and extreme weather affect normal crop growth and development, causing massive yield losses and quality degradation [1, 2]. Through continuous evolution, a series of mechanistic changes have occurred in plants at the molecular, physiological, and biochemical levels to maximize resistance to the adverse effects of abiotic stressors [3]. For example, transcription factors (TFs) and protein kinases can regulate the downstream signaling pathways that activate and regulate specific stress-related genes, ultimately leading to physiological responses to stress such as accumulation of osmoregulatory substances [4, 5]. Specific functional proteins related to stress tolerance in plants, for example, late embryogenesis abundant (LEA) proteins that are highly enriched in late embryogenesis and nutritional tissues in response to water deficit, can eliminate reactive oxygen species (ROS) from cells to protect macromolecules and mitigate the damage caused by abiotic stressors [6, 7].

LEA proteins is the collective term for a large group of glycine-rich, hydrophilic, and structurally intrinsically disordered proteins that are widely distributed in the plant kingdom [8]. A study in 1981 first identified a LEA protein in *Gossypium hirsutum* and showed that it accumulates in large quantities during seed dehydration and maturation to protect the seed from damage [9]. Subsequently, with the development of whole-genome sequencing technology, additional LEA proteins were identified in model crops (*Arabidopsis thaliana* and *Oryza sativa*) [10, 11], food crops (*Zea mays* and *Triticum aestivum*) [12, 13], horticultural crops (*Cucumis sativus* and *Vitis vinifera*) [14–16], and other species (microbes and invertebrates) [17]. Most LEA proteins, except for a small proportion in the endoplasmic reticulum of plant cells, are highly diverse and are distributed in the nucleus, cytoplasm, and mitochondria [18]. The classification of LEA proteins differs among species. Typically, LEA proteins are classified into eight different clusters based on sequence similarity, specific motifs, and conserved structural domains [19, 20].

As LEA proteins have unique biochemical properties, such as high hydrophilicity [21], presence of a large number of charged amino acid (AA) residues [22], glycine, or other tiny AAs [23], small number of cysteine residues [24], and a lack of tryptophan residues [7], they are tolerant to heat and acid and participate in numerous physiological processes associated with plant development and responses to adversity, thereby contributing to various stress tolerance responses [7, 20, 25].

It is widely known that an extremely rapid increase in the cytosolic ion concentration can cause irreversible cell damage in plants subjected to adversity [26]. LEA proteins with bound divalent cations can redirect water molecules in cells, bind salt ions, and activate abiotic stress-induced oxidative scavengers in cells [27]. LEA proteins effectively slow down the inactivation of malate dehydrogenase, lactate dehydrogenase, catalase, citrate synthase, and some mitochondrial enzymes in response to water deficiency and exhibit good protective properties against enzymatic activity [28–30]. A few LEA proteins act by stabilizing the membranes; sugar-bound membrane maintenance and ion segregation help in preventing the collapse of cellular structures [6, 31, 32]. Furthermore, LEA proteins can repair misassembled proteins to recover their biological activity by utilizing molecular chaperones that bind to misfolded proteins [33, 34].

The imperative role of LEA proteins in plant reactions to abiotic stressors has been reported in many studies. Overexpression of *AtLEA14* overactivates the transcripts of salt stress response marker genes in transgenic *A. thaliana*, which exhibits high salt tolerance [35]. Overexpression of *OsLEA3-2* increases salt and drought tolerance in transgenic *A. thaliana* and *O. sativa* [36]. During the nutritional growth period, *OsEm1* responds to the hormone abscisic acid (ABA) and abiotic stressors, and overexpression of *OsEm1* increases the survival rate and expression of other *LEA* genes in transgenic *O. sativa*, thereby improving its sensitivity to ABA and enhancing tolerance to osmotic stressors [37]. Both abiotic stress and signaling molecules induce *ZmLEA3* expression in leaves, and overexpression of *ZmLEA3* improves the tolerance of transgenic *Nicotiana tabacum* to osmotic and oxidative stress [38]. In addition, the *CaLEA1* gene in *Capsicum annuum* [39], *TaLEA3* gene in *Leymus chinensis* [40], *IpLEA* gene in *Ipomoea pes-caprae* [41], *SmLEA* gene in *Salvia miltiorrhiza* [42], and *SiLEA14* gene in *Setaria italica* [43] have also been reported to play roles in stress tolerance.

*S. lycopersicum* is an important vegetable crop and a model plant belonging to the Solanaceae family. However, its growth and development, yield, and quality are severely constrained by abiotic stressors [44]. Conversely, wild tomato species have become tolerant to abiotic stressors and have been able to grow normally in extreme environments after differential expression of different genes activated certain molecular and physiological mechanisms that allowed them to adapt effectively [45, 46]. Previous genome sequencing-based research on *S. lycopersicum*, *Solanum pimpinellifolium*, *Solanum pennellii*, and *Solanum lycopersicoides* provides an excellent opportunity to identify and analyze the *LEA* gene family in the context of abiotic stress [47, 48].

In the present study, we aimed to perform genome-wide identification of *LEA* genes in the above-mentioned tomato species and systematically analyze the phylogeny, gene structure, conserved structural domains, motifs, chromosome distribution, purification pressure, duplication date, tandem duplication, and segmental duplication events. We also conducted homology and synteny analyses among different species, analyzed *cis*-acting regulatory elements, transcriptional expression profiles, expression patterns under different abiotic stressors and phytohormone treatments, gene ontology (GO) annotation, protein interaction networks, and performed virus-induced gene silencing (VIGS) to provide new information regarding the *LEA* gene family and a reference for future molecular and biofunctional studies aimed at developing genetic improvement strategies in tomato.

## Results

### *LEA* identification and characterization

We used the protein families (Pfam) database identity documents (IDs) (PF03760, PF03168, PF03242, PF02987, PF0477, PF10714, PF04927, and PF00257) from *S. lycopersicum*, *S. pimpinellifolium*, *S. pennellii*, and *S. lycopersicoides,* which were identified as having 60, 69, 65, and 60 *LEA* genes, respectively. The identified LEA genes were divided into eight clusters—LEA_1 (18 genes), LEA_2 (160 genes), LEA_3 (16 genes), LEA_4 (12 genes), LEA_5 (7 genes), LEA_6 (2 genes), dehydrin (25 genes), and seed maturation protein (SMP) (14 genes). The largest and smallest numbers of *LEA* genes were observed in the LEA_2 and LEA_6 clusters, respectively. All LEA proteins were predicted to have different physicochemical properties; the peptide length ranged from 78‒737 AA, with a mean of 228.44 AA. The molecular weight (MW) of these 254 LEA proteins ranged from 8.87‒78.60 kDa. The isoelectric point (pI) ranged from 4.42‒10.42, with 25.20% being acidic proteins (pI < 7) and 74.80% being basic proteins (pI > 7). The instability coefficients ranged from 6.65‒59.1, with 53.94% of LEA proteins having low instability index (< 40), indicating that > 50% of the LEA proteins were stable at the theoretical level. The grand average of the hydropathy index (GRAVY) suggested that > 83.46% of LEA proteins were hydrophilic (GRAVY < 0). We observed similar physicochemical parameters for most *LEA* genes in the same family, which was in agreement with the main characteristics of *LEA* genes (i.e., low hydrophobicity and high net charge, which can help plants to resist desiccation effects). The predicted possible subcellular localization of all *LEA* genes revealed that they had a broad distribution at the cellular level, with the majority targeting the nucleus (39.37%) or plasma membrane (26.77%). Contrastingly, some other members were localized in cellular structures such as the cytoplasm, extracellular matrix, mitochondria, and chloroplasts, thus establishing protective mechanisms and enhancing intermembrane interactions to effectively respond to various external stressors (Additional file 1: Table S1).

### Phylogeny, gene structure, conserved domains, and motif analysis of *LEA* genes

**Fig. 1:**
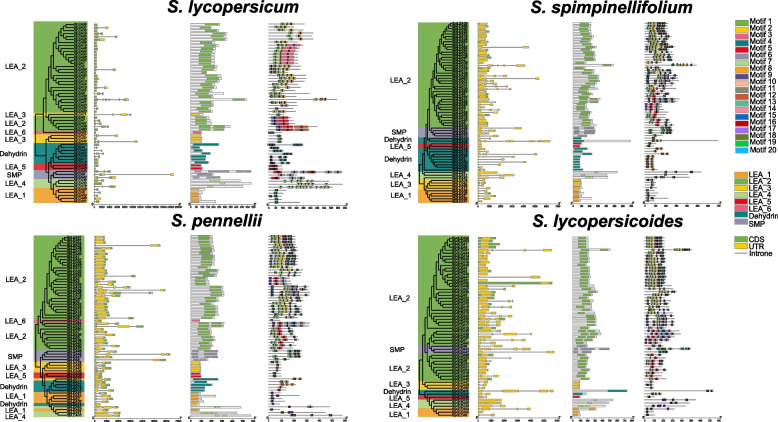
Phylogeny, gene structure, conserved domains, and motifs of *LEA* genes in cultivated and wild tomato species. Each diagram represents, in order from left to right, the phylogenetic tree of LEA proteins; structure of exons, introns, and untranslated regions (UTR) in *LEA* genes; distribution of conserved domains of LEA proteins; different motifs present in LEA proteins

To investigate the homology and similarity, unrooted phylogenetic trees were created based on the comparison of LEA protein sequences of cultivated and wild tomato species (Fig. 1). Phylogenetic analysis indicated that they were separated into eight clusters belonging to two main branches; generally, the LEA_2 cluster was in one branch and the other clusters were in the other branch. Furthermore, the phylogeny of *LEA* genes exhibited strong correlations with gene structure, conserved domain, and motif. Genetic structure analysis revealed that the introns and exons of *LEA* genes had different structural features and that the most closely related *LEA* genes in the same group had similar gene structures in terms of intron number and exon length. The majority of genes had intron numbers ranging from 0‒3. Ninety-six of the genes had no introns, which implied that the *LEA* genes may have lost numerous introns during their evolution. Conserved domain analysis showed that except for SMP, the other LEA protein subfamily members had a simple structure, mostly containing a LEA structural domain; most of these were near the C-terminus of the protein, with a few at the N-terminal or intermediate position. The same LEA subfamily contained the same protein structure domain, suggesting that their main functions may be the same. Our further conserved motif analysis showed that among the 20 different motifs, motifs 1, 2, 3, and 10 were common in the LEA_2 subfamily of cultivated and wild tomato species. Except for the LEA_2 subfamily, the other LEA subfamily members had a relatively simple motif composition, mostly comprising 1‒3 motifs. Each LEA subfamily member contained several identical motifs specific to the group; this taxon specificity implied that the members belonging to the same taxon have similar biological functions. The formation of motif patterns suggested that LEA proteins are positively engaged in a multitude of biological processes (BP). The diverse nature of the conserved motifs suggested that LEA proteins evolved from the amplification of genes within their particular gene families.

### Phylogenetic tree analysis of LEA proteins

**Fig. 2:**
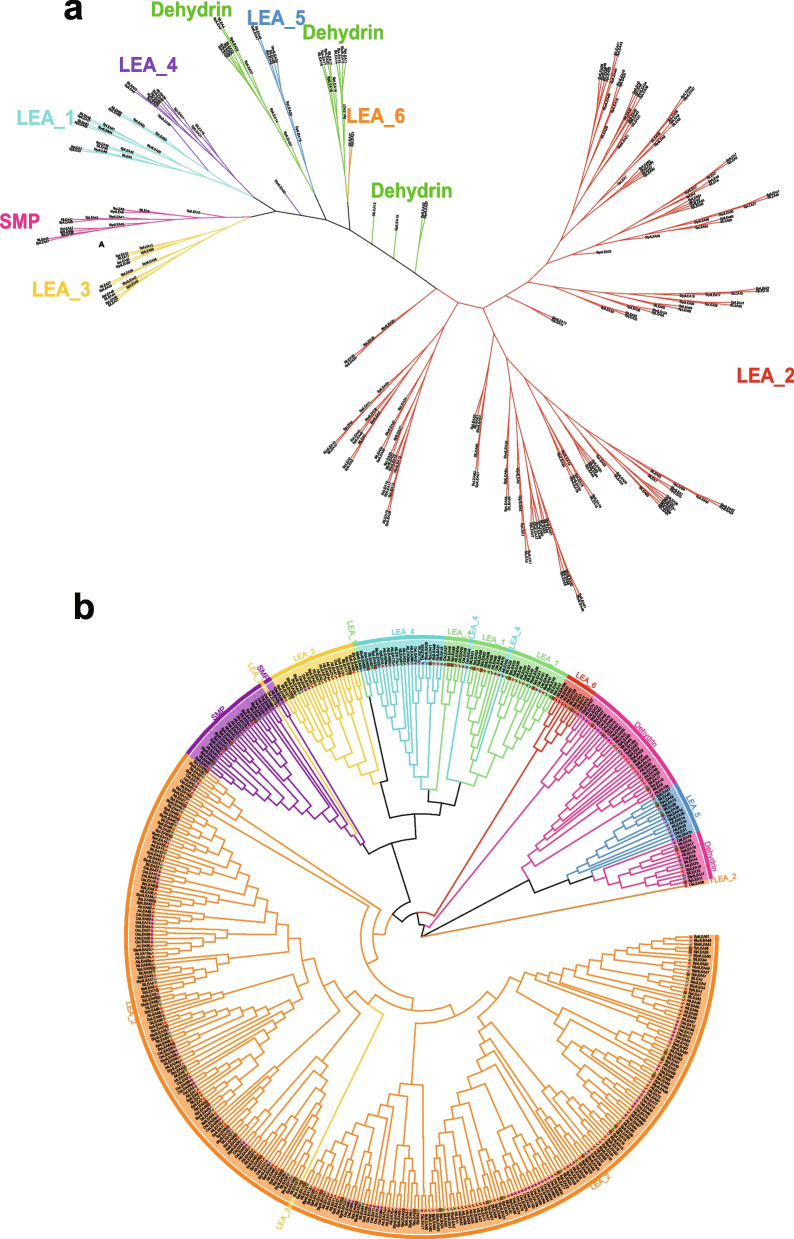
Phylogenetic tree analysis of LEA proteins. **a** Phylogenetic tree of LEA proteins between cultivated and wild tomato species. **b** Phylogenetic tree of LEA proteins between tomato and other species. The sequences of all LEA proteins were compared with multiple sequences, and a phylogenetic tree was constructed using the maximum likelihood method and 1000 bootstrap resampling in MEGA 11 software. Each color represents a cluster

Considering the large differences in length and different structural domains of the LEA protein family members, we first performed a phylogenetic tree analysis of all 254 LEA proteins between cultivated and wild tomato species (Fig. 2a). The results showed that they were mainly divided into eight evolutionary branches, with the LEA_2 subfamily at one end of the main branch and the remaining subfamilies at the other end of the main branch. Except for the dehydrin subfamily (which was divided into five sub-branches), the other branches included the corresponding LEA subfamily members. Subsequently, we performed a phylogenetic analysis of all LEA proteins with the 97 LEA proteins present in the *A. thaliana* and *O. sativa* genomes (Fig. 2b), which we re-predicted and identified. Previous studies predicted that the *A. thaliana* [10] and *O. sativa* [11] genomes contain 51 and 35 LEA proteins, respectively; because of the large differences, in this study they were renamed according to the order of their distribution from top to bottom in each chromosome (further information is provided in Additional file 2: Table S2). This enabled comprehension of the evolutionary relationships of LEA families among different model plants. The results of phylogenetic tree analysis showed that the main evolutionary branches corresponded to different LEA subfamilies and that each subfamily could be divided into two subfamilies. In general, the LEA_2 subfamily contained the most members, the LEA_6 subfamily contained the least members, and the members in the same subfamily had similar origins and evolutionary relationships. The different subfamilies contained LEA members from *O. sativa*, *A. thaliana*, and cultivated and wild tomato species, and their origins presumably appeared before the divergence of monocotyledons and dicotyledons.

### Chromosome distribution, duplication events, purification pressure, and synteny analysis

**Fig. 3:**
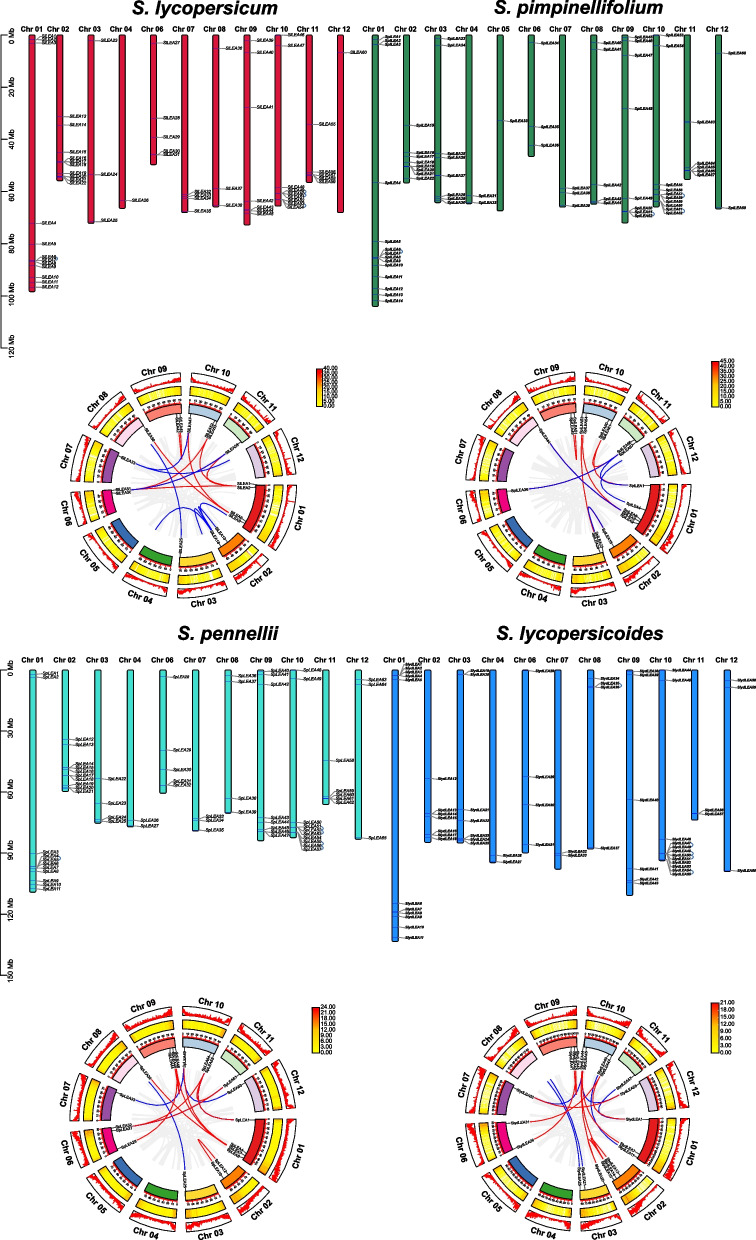
Chromosomal localization and gene duplication events of *LEA* genes in cultivated and wild tomato species*. LEA* distribution on the chromosomes of cultivated and wild tomato species is represented by different colored bars; each blue arc represents a pair of tandem duplicated genes. The different colored lines in the corresponding circle plots indicate synthetic pairs of *LEA* genes, and the gray lines indicate synthetic pairs of all genes

The genomic distribution of *LEA* genes varied depending on the chromosome (Fig. 3). They were distributed on all chromosomes of *S. pimpinellifolium* and all chromosomes of *S. lycopersicum*, *S. pennellii*, and *S. lycopersicoides* except for chromosome 5. Among *S. lycopersicum*, *S. pimpinellifolium*, *S. pennellii*, and *S. lycopersicoides*, all *LEA* genes were most densely distributed on chromosome 1, with 12, 14, 11, and 11 members, respectively. In contrast, they had a sparse distribution on chromosomes 4 and 12, on which only 1‒3 members were distributed. Most *LEA* genes were distributed at both ends of the chromosomes. An analysis of intra-genomic duplication events in all *LEA* genes showed that *S. lycopersicoides* (located on chromosomes 8 and 10) produced five pairs of tandem duplication events, and *S. lycopersicum*, *S. pimpinellifolium*, and *S. pennellii* (located on chromosomes 1, 9, and 10) produced four, four, and six pairs of tandem duplication events, respectively (Fig. 3, Additional file 3: Table S3).

Furthermore, we found that *S. lycopersicum*, *S. pimpinellifolium*, *S. pennellii*, and *S. lycopersicoides* exhibited seven, seven, nine, and seven pairs of segmental duplication events distributed on the corresponding chromosomes, respectively (Fig. 3, Additional file 3: Table S3). Segmental duplication events were more pronounced than tandem duplication events, which may imply that segmental duplication events were a major contributor to the evolutionary process.

We first assessed species selection and evolution using the non-synonymous substitution (*K*a)/synonymous substitution (*K*s) ratio. The ratios of tandem duplication events in *LEA* genes ranged from 0.08 to 2.59, with a mean of 0.83. The ratios of segmental duplication events ranged from 0.18 to 2.20, with a mean of 0.98 (Additional file 3: Table S3). The mean values of the ratios of all segmental duplication and tandem duplication gene pairs were < 1, suggesting that *LEA* genes were subjected to purifying selection during evolution. We then used the *K*s values to predict the dispersion time of *LEA* duplication events. Tandem duplication of *LEA* genes occurred between 3.17 and 92.64 million years ago (MYA), and segmental duplication occurred between 31.18 and 149.38 MYA (Additional file 3: Table S3).

We also discovered that seven pairs of segmental duplication events occurred between 40 and 50 MYA. Furthermore, we predicted that the tandem duplication events of the *SpLEA55/SpLEA56*, *SlLEA53*/*SlLEA54*, and *SlydLEA50/SlydLEA51* gene pairs occurred at approximately 3.17, 3.33, and 3.94 MYA, i.e., after the tomato–*Solanum tuberosum* division [49].

**Fig. 4:**
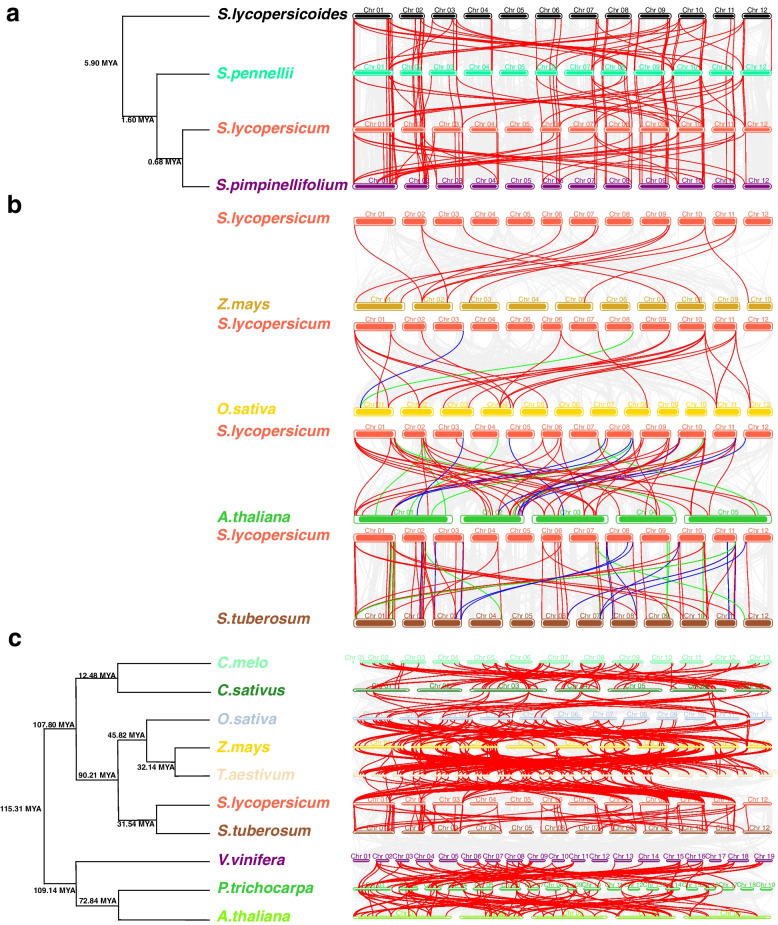
Synteny plot of *LEA* genes. **a** The red line indicates the synteny of *LEA* genes in cultivated and wild tomato species. **b** Synteny plot of *SlLEA* genes with those of the other four species. Gray lines indicate synteny between the *S. lycopersicum* genome and those of other species. Red lines indicate synteny between *SlLEA* genes and the *LEA* genes of other species. Blue lines indicate synteny between *SlLEA* genes and the non-*LEA* genes of other species. Green lines indicate synteny between *LEA* genes of other species and non-*LEA* genes of *S. lycopersicum*. **c** Red lines indicate synteny of *LEA* genes among the 10 species

By constructing divergence time trees and synteny profiles between cultivated and wild tomato species, we gained further insights into the divergence time and synteny relationships of *LEA* genes (Fig. 4a). The results showed that they were mainly divided into two evolutionary branches at around 5.9 MYA, with *S. lycopersicum* and *S. pimpinellifolium* having similar divergence times at around 0.68 MYA; contrastingly, their divergence times with respect to other species were farther apart. Similar synteny relationships among *LEA* genes existed between cultivated and wild tomato species. Most of them formed approximately three synteny gene pairs, which suggested a conserved evolutionary role of the *LEA* family in cultivated and wild tomato species.

We further elucidated the sequence similarity of *S. lycopersicum LEA* (*SlLEA*) genes with related *LEA* genes and homologous genes of other species; to this end, we constructed synthetic maps of *SlLEA* genes with the *LEA* genes of two monocots (*Z*. *mays* and *O. sativa*) and two dicotyledons (*A. thaliana* and *S. tuberosum*) (Fig. 4b). The outcome revealed that the synteny profiles of *SlLEA* genes were largely consistent with those of the remaining four species. We identified 14, 27, 47, and 57 repeat events (shown as red lines) in *Z*. *mays*, *O. sativa*, *A. thaliana*, and *S. tuberosum*, respectively. We found no synteny between *S. lycopersicum* and *Z*. *mays* and *O. sativa* on chromosomes 4, 5, and 12; synteny modules were mainly distributed on the remaining chromosomes. *S. lycopersicum* had synteny modules with *S. tuberosum* except for chromosome 5, whereas *S. lycopersicum* had synteny modules with *A. thaliana* on all chromosomes. *SlLEA* genes had no homologous genes in *Z*. *mays*. *SlLEA38* on *S. lycopersicum* chromosome 8 was homologous to *Os01t0234700*-*01* on *O. sativa* chromosome 1 (shown as green lines), and *OsLEA4* on *O. sativa* chromosome 1 was homologous to *Solyc03g120720.3.1* on *S. lycopersicum* chromosome 3 (shown as blue lines). There were multiple pairs of homologous genes between *S. lycopersicum* and *A. thaliana* and between *S. lycopersicum* and *S. tuberosum*, with *SlLEA18* and *SlLEA48* being homologous in both *A. thaliana* and *S. tuberosum*. Similarly, *SlLEA38* had homologous genes in both *O. sativa* and *A. thaliana*.

Analyses of divergence times and syntenic relationships of *LEA* genes among the other 10 species showed that they mainly divided into two evolutionary branches around 108 MYA, with tomato and *S. tuberosum* having similar divergence times to rice and maize at approximately 32 MYA (Fig. 4c). More synteny relationships existed among *LEA* genes in monocotyledons (*O. sativa*, *T. aestivum*, and *Z. mays*). Except for *S. tuberosum* and *V. vinifera*, synthetic relationships existed between the *LEA* genes of adjacent species. Some *LEA* genes formed 2‒5 synteny gene pairs, which may have played an important role in the evolution of the *LEA* gene family, and indirectly suggested that the structure and function of *LEA* genes are variable and diverse.

### Analysis of *cis*-acting regulatory elements of *LEA*

**Fig. 5:**
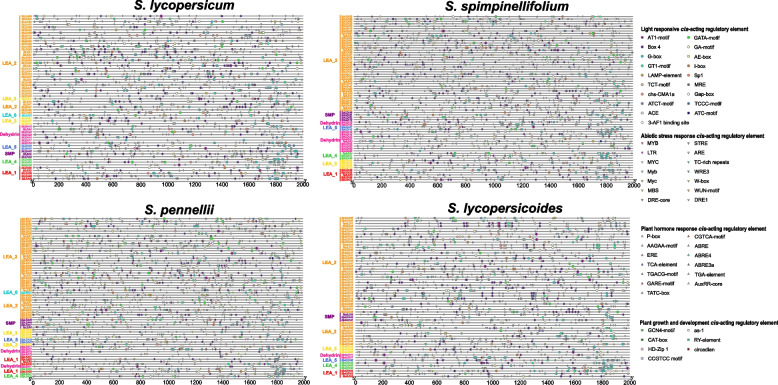
Predicted*cis*-acting regulatory elements in the promoter regions of *LEA* genes in cultivated and wild tomato species. Gene names are arranged according to the order of the phylogenetic tree. Different colors and shapes of patterns represent different classes of *cis*-acting regulatory elements

Our prediction analysis of *cis*-acting regulatory elements at the 2000 bp nucleotide sequence upstream (from ATG) of each *LEA* in cultivated and wild tomato species showed that except for the common *cis*-acting regulatory elements, i.e., TATA-box and CAAT-box, in the promoter region, the other *cis*-acting regulatory elements present could be classified into four categories (Fig. 5). The first category was light-responsive *cis*-acting regulatory elements; all *LEA* genes in cultivated and wild tomato species contained 19 such *cis*-acting regulatory elements (including AT1-motif, GATA-motif, GA-motif, Box4, and G-box), and almost all *LEA* genes comprised at least one light-responsive *cis*-acting regulatory element in their promoter regions. The second category was *cis*-acting regulatory elements related to responses to abiotic stress environments or external stressors. Thirteen such *cis*-acting regulatory elements (including MYB, STRE, LTR, ARE, and MYC) suggested that *SlLEA* genes may play key roles in abiotic stress defense and stress, drought response, and anaerobic induction. In addition, some *LEA* genes contained W-box motifs, which were combining locus for *WRKY* TFs. The third category was phytohormone-responsive *cis*-acting regulatory elements. Thirteen such *cis*-acting regulatory elements, including P-box, CGTCA-motif, AAGAA-motif, ABRE, and ERE, were related to the response to various hormones such as ABA, methyl jasmonate (MeJA), salicylic acid, gibberellins (GA), and auxin (IAA), respectively. The fourth category of *cis*-acting regulatory elements was involved in plant growth and development (such as GCN4-motif, as-1, and CAT-box) and included seven such *cis*-acting regulatory elements, which were associated with growth and developmental processes such as tomato meristem expression, chloroplast differentiation, and endosperm expression.

### Analysis of the expression pattern and transcriptional profiles of *LEA* genes

**Fig. 6:**
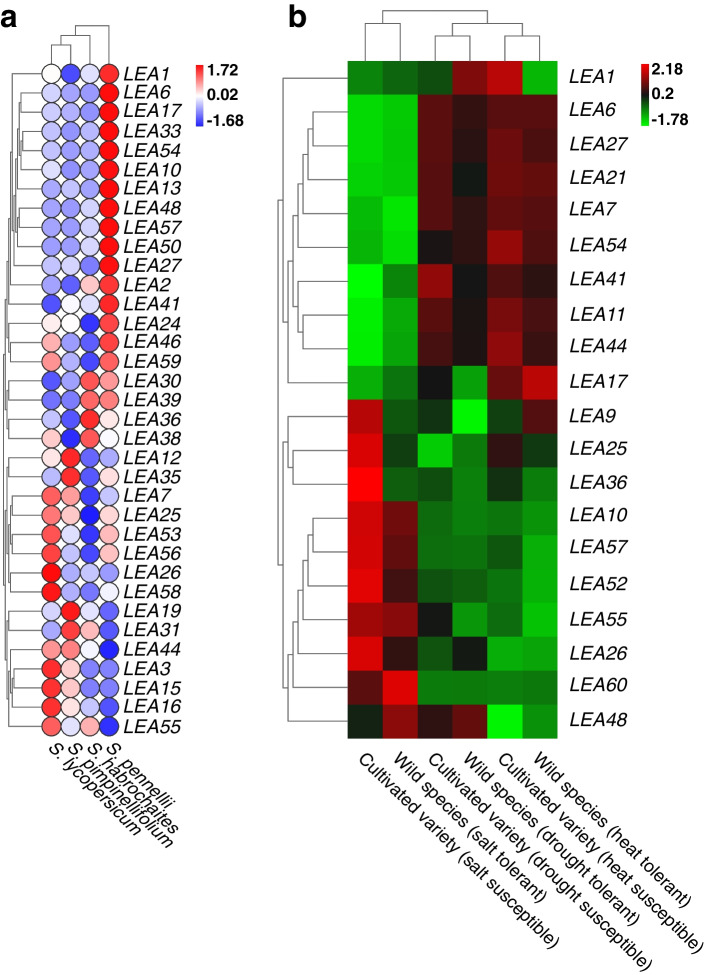
Clustering heat map of *LEA* genes from leaves of cultivated and wild tomato species under (**a)** normal and (**b**) abiotic stress conditions. Salt stress: *S. lycopersicum* (salt-susceptible cultivar) versus *S. pimpinellifolium* (salt-tolerant wild tomato species) (GEO accession number GSE16401). Plants were treated with 200 mM NaCl for 5 h. Drought stress: *S. lycopersicum* (drought-susceptible cultivar) versus *S. habrochaites* (drought-tolerant wild tomato species) (GEO accession number GSE22304). Plants were subjected to water stress by holding water for 7 d. Heat stress: *S. lycopersicum* (heat-susceptible cultivar) versus *S. habrochaites* (heat-tolerant wild tomato species) (GEO accession no. GSE22304). Plants were treated at a high temperature of 40 ℃ for 1 h

We first analyzed the expression profiles of *LEA* genes in *S. lycopersicum*, *S. pimpinellifolium*, *S. habrochaites*, and *S. pennellii* leaves in an attempt to reveal the differential regulation patterns between cultivated and wild tomato species under normal conditions (Fig. 6a). The clustered heat map results showed that 16 *LEA* genes, including *LEA1*/*6*/*17*, were significantly overexpressed in *S. pennellii*, *LEA30*/*36*/*38*/*39* were significantly overexpressed in *S. habrochaites*, *LEA12*/*19*/*31*/*35* were significantly highly expressed in *S. pimpinellifolium*, and *LEA3*/*15*/*16*/*26*/*58* were significantly highly expressed in *S. lycopersicum*. Different patterns of differential regulation were observed between cultivated and wild tomato species under normal conditions; this up and downregulation relationship of *LEA* genes among different cultivated and wild tomato species may also have been closely related to their abiotic stress tolerance-related expression patterns.

To obtain more information on the role of *LEA* genes under abiotic stress conditions, we analyzed their transcriptional expression profiles under salt, drought, and high-temperature stress and compared the results to those of the wild species (Fig. 6b). Under salt stress, *LEA9/25/26/36/52* were downregulated in wild species, whereas *LEA48/60* were upregulated in wild species compared to those in cultivars. Under drought stress, *LEA11/21/41/44* were downregulated in the wild species, whereas *LEA1/48/54* were upregulated in the wild species compared to those in cultivars. Under high-temperature stress, *LEA1/25/44/54* were downregulated in the wild species, whereas *LEA9/17* were upregulated in the wild species compared to those in cultivars. *LEA9* was downregulated under salt stress and upregulated under high-temperature stress compared to that in the cultivar. *LEA1/54* were upregulated under drought stress and down-regulated under high-temperature stress compared to those in the cultivar. *LEA25* was downregulated under both salt and high-temperature stress compared to that in the cultivar, *LEA44* was downregulated under both drought and high-temperature stress compared to that in the cultivar, and *LEA48* was upregulated under both salt and drought stress compared to that in the cultivar. The differential up or downregulation of *LEA* genes under different stress conditions indicated their different patterns of regulation in cultivated and wild tomato species, which are complex and diverse.

**Fig. 7:**
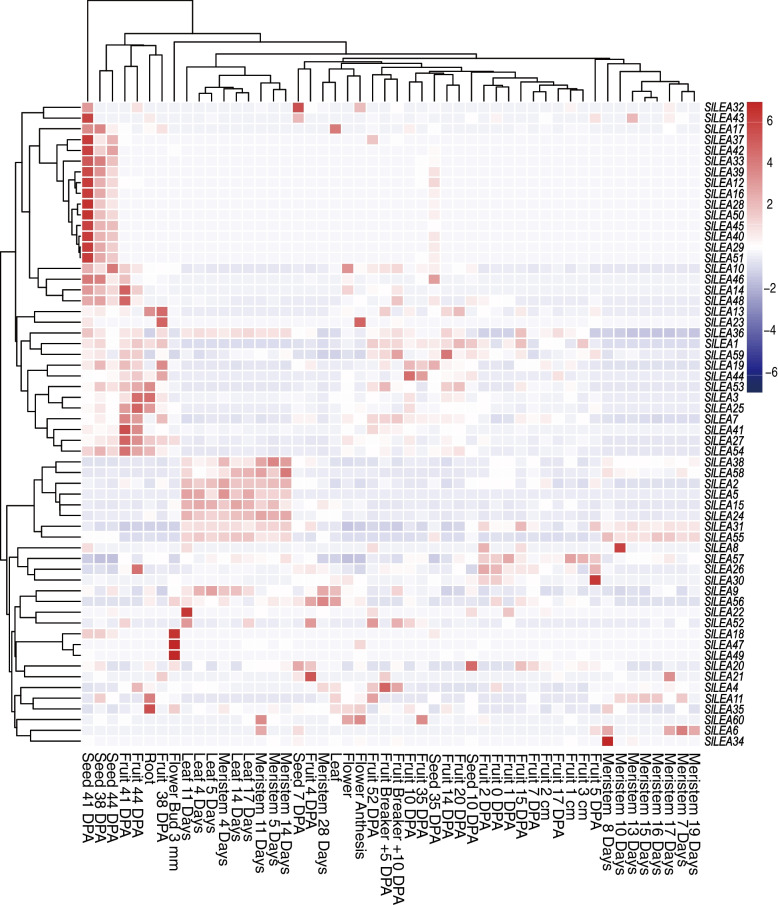
Clustering heat map of *SlLEA* expression patterns in various tissues. Horizontal coordinates indicate the seeds, roots, meristems, leaves, flowers, and fruits of cultivated tomatoes at different growth periods. Developmental stages include the seed (7, 10, 35, 38, 41, and 44 DPA), root, meristem (4, 5, 7, 8, 10, 11, 13, 14, 15, 16, 17, 19, and 28 d), leaf (4, 5, 11, 14, and 17 d), flower (anthesis and bud 3 mm), and fruit (0, 1, 2, 4, 5, 7, 10, 14, 15, 17, 20, 35, 38, 41, 44, and 52 DPA; 1, 2, and 3 cm; breaker + 5 and + 10). Vertical coordinates indicate *SlLEA* expression pattern. The color gradient from blue to red indicates low to high expression

To further explore the potential features of *SlLEA* genes, the transcript profile expression values of each *SlLEA* on the TomExpress online platform were systematically analyzed. The expression patterns of *SlLEA* genes in nutritional and reproductive tissues were presented in the form of an additional clustered heat map (Fig. 7). *SlLEA* genes were expressed in several examined tissues, and the expression levels varied considerably among tissues and periods. *SlLEA* genes were generally underexpressed in meristematic tissues, leaves, and flowers and generally overexpressed in seeds and fruits. Among them, certain genes exhibited unique expression profiles. For example, *SlLEA20* showed the highest expression during mid-seed development (10 days post anthesis, DPA), *SlLEA35* in roots, *SlLEA8* during mid-meristem tissue development (10 DPA), *SlLEA22* during mid-leaf development (11 d), and *SlLEA34* during early flower development (8 d). *SlLEA2/5/15/24/31/38/55/58* were consistently expressed at high levels during leaf and phloem tissue growth and development. *SlLEA18/47/49* showed the highest expression in flower buds (3 mm). *SlLEA26/30/57* were consistently highly expressed during the initial periods of fruit development (0, 1, 2, and 5 DPA). More than half of the *SlLEA* genes were persistently highly expressed during late seed development (38, 41, and 44 DPA), which suggested a potential role of these genes in regulating seed maturation. *SlLEA1/3/7/13/36/53/54/59* showed high expression levels during the middle and late periods of fruit development, which suggested that they may be engaged in the growth and development of *S. lycopersicum* fruits (from immature to mature). The above-mentioned analysis of *SlLEA* expression pattern provides additional insights into their possible roles.

### Analysis of *SlLEA* expression patterns in response to abiotic stressors and phytohormone treatments

We identified the *cis*-acting regulatory elements of all *LEAs* that may be responsible for directing their differential activation or repression concerning an abiotic stress environment or external stress and phytohormone responses and analyzed the expression patterns and transcriptional profiles of *SlLEA* genes. Following this, we randomly selected 24 *SlLEA* genes in an attempt to identify the genes contributing to responses to various abiotic stressors and phytohormone treatments. General expression characteristics of *SlLEA* genes in response to different abiotic stressors and phytohormone treatments were examined to better understand the possible roles of various *SlLEA* family members.

In the present analysis, we only considered genes with a > fivefold changes in transcript abundance and *P-*values < 0.05. Using this criterion, we observed that none of the *SlLEA* genes showed a significant change in expression at any period under the control treatment in normal conditions (Additional file 4: Figure S1). However, under simulated drought stress, *SlLEA4/6/12/14/17/27/50* were significantly upregulated at the early stage (1 or 3 h) and maintained a high expression profile until the middle stage (12 h). Among them, *SlLEA6* and *SlLEA17* were rapidly upregulated by approximately 50-fold at the initial (1 h) and middle (12 h) stages, respectively (Additional file 5: Figure S2). Under simulated high salt stress, *SlLEA4/6/12/14/16/17/18/27/33/50* were significantly upregulated at the early stage (1 or 3 h) and maintained high expression until the late stage (24 h). Among them, *SlLEA6/50/17* were strongly upregulated by more than 100-fold in the initial (1 h), middle (12 h), and late (24 h) stages, respectively (Additional file 6: Figure S3). Under simulated high-temperature stress, *SlLEA27/37/50/60* responded rapidly and were significantly upregulated at the early stage (1 or 3 h); *SlLEA60* maintained high expression from the initial (1 h) to the late (24 h) stage, and *SlLEA37* was rapidly upregulated by more than 60-fold at the initial stage (1 h) (Additional file 7: Figure S4). Under simulated low-temperature stress, *SlLEA6/14/27* were significantly upregulated at the early stage (1 or 3 h) and maintained high expression until the late stage (24 h); *SlLEA50* was significantly upregulated at the middle and late stages (12 h and 24 h), and *SlLEA6* was rapidly upregulated by more than 60-fold at the middle stage (12 h) (Additional file 8: Figure S5).

*SlLEA* genes showed no significant changes in expression at any period under the control treatment of ddH_2_O spraying (Additional file 9: Figure S6). Contrastingly, under the phytohormone ABA treatment, *SlLEA6* and *SlLEA27* were rapidly induced and their expression was highly significantly increased at the initial stage (1 h). *SlLEA6* was rapidly upregulated by approximately 150-fold, and *SlLEA14* expression was highly significantly increased at the middle stage (12 h) (Additional file 10: Figure S7). Under the phytohormone MeJA treatment, *SlLEA6* was rapidly induced and highly significantly upregulated at the initial (1 h) and middle (6 h) stages, *SlLEA4* and *SlLEA17* were highly significantly upregulated at the late stage (24 h), and *SlLEA27* maintained high expression from the early (1 h) to the late (24 h) stage (Additional file 11: Figure S8). Under the strigolactones (*rac*-GR24) treatment, *SlLEA6* was rapidly induced at the early stage (1 h), was highly significantly up-regulated, and maintained high expression until the middle stages (6 h). *SlLEA27* responded rapidly at the early stage (1 h), was highly significantly upregulated, and maintained high expression until the middle stage (12 h) (Additional file 12: Figure S9). *SlLEA7* was rapidly induced and highly significantly up-regulated at the initial stage (1 h) upon treatment with the phytohormone γ-aminobutyric acid (GABA). *SlLEA6/16/27* responded rapidly and were significantly upregulated at the early stage (1 h and 3 h), with *SlLEA6* being strongly upregulated by more than 80-fold at the initial stage (1 h) (Additional file 13: Figure S10). The different expression patterns of these *SlLEA* genes suggested that they performed particular functions under different stressor and phytohormone treatments. Overall, *SlLEA* expression was more sensitive to high salt and drought stressors than temperature. Some *SlLEA* genes showed similar expression profiles in response to various abiotic stressors; *SlLEA4/12/17* responded to both high salt and drought stressors; *SlLEA6/14/50* responded to high salt, drought, and low-temperature stressors; *SlLEA27* simultaneously responded to all four of these stress conditions. Similarly, *SlLEA6* and *SlLEA27* exhibited comparable expression patterns in response to different phytohormone treatments and simultaneously responded to the four phytohormone treatments. *SlLEA6* simultaneously responded to all of the conditions mentioned above except high-temperature stress, and *SlLEA27* simultaneously responded to all of the conditions mentioned above. These findings indicate that *SlLEA6* and *SlLEA27* may be the key genes in the *SlLEA* family that participate in defense and stress responses to external stressors.

### GO annotation and enrichment analyses

We first investigated the BP, molecular functions (MF), and cellular components (CC) of *LEA* genes in cultivated and wild tomato species. The GO analysis results indicated that 254 *LEA* genes may be involved in a range of BP. Among them, most *LEA* genes were predicted to play roles in the responses to abiotic stimuli and water deficit. The prediction of MF indicated that most *LEA* genes were involved in single-stranded DNA binding and transferase activity. Genes involved in condensed nuclear chromosome and egg chorion were higher in the predictions for CC (Additional file 14: Figure S11).

**Fig. 8:**
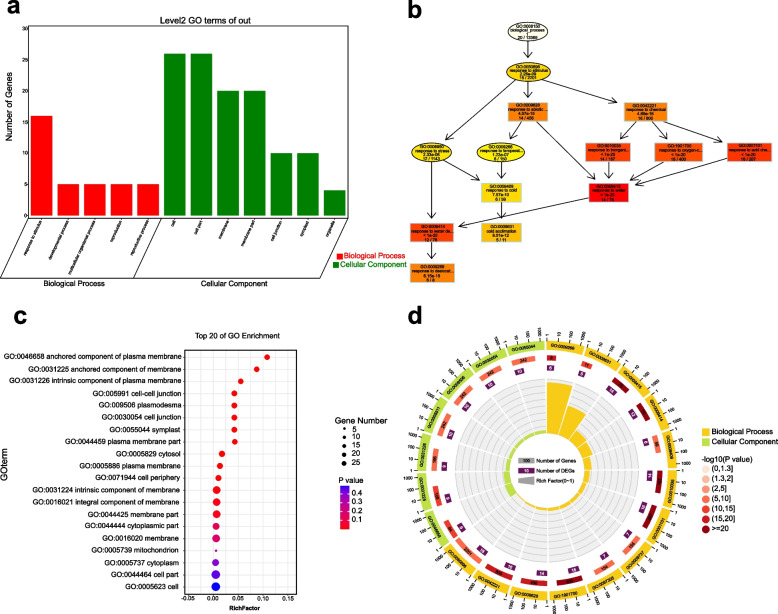
GO annotation and enrichment analyses of *SlLEA* genes. **a** Statistical graph of the secondary classification of GO. Horizontal coordinates represent the secondary classification of GO ontology, and vertical coordinates represent the number of genes present in each classification entry, thus indicating the number and enrichment of target genes in each GO term. **b** Directed acyclic graph of GO. The GO term closer to the root node is more general, and the GO term branching down is annotated to a finer level. The first 10 GO terms with the highest salience are rectangular, and the other GO terms are circular. The darker the color, the more significant the GO term, from light to dark: no color, light yellow, dark yellow, and red, respectively. **c** Significant bubble plot. The top 20 significantly enriched GO terms are displayed by default. The color represents the *P*-value, the bubble size represents the number of genes enriched to this GO term, and the rich factor in the horizontal coordinate of the bubble plot represents the ratio of the number of differentially expressed genes located in this GO term to the total number of genes located in this GO term among all annotated genes (i.e., the larger the ratio, the greater the enrichment). **d** Enrichment circle map. The first circle is the enriched classification, and the outside of the circle is a coordinate scale for the number of genes, with different colors representing different classifications. The second circle shows the number of that classification in the background genes and the *P*-value, whereby the more genes the longer the bar and the smaller the value the redder the color. The third circle shows the total number of foreground genes. The fourth circle shows the rich factor value for each classification (i.e., the number of foreground genes in that classification divided by the number of background genes), with each small cell of the background auxiliary line indicating 0.1

We further performed GO annotation and enrichment analyses of 60 *SlLEA* genes to gain a more in-depth understanding of the functions of these genes. The GO secondary classification statistical graph results indicated that only two major categories (i.e., BP and CC) were observed in GO terms, with no MF (Fig. 8a). The GO-directed acyclic graph confirmed that these *SlLEA* genes were involved in a range of BP; most *SlLEA* genes were predicted to play a role in the responses to desiccation and water deficit and to respond to abiotic stimuli and stressful environments (Fig. 8b). The GO significance bubble plots indicated that the CC of *SlLEA* genes was predicted to be the most functionally enriched, involving the membrane, cell, and cytoplasmic fractions (Fig. 8c). The innermost layer of the enrichment circle plot was particularly visually focused to show the rich factor values (i.e., the number of foreground genes divided by the number of background genes) for each taxon, including response to desiccation (GO: 0,009,269) (6/8), cold acclimation (GO: 0,009,631) (5/11), and response to water (GO: 0,009,415) (14/78) (Fig. 8d), further suggesting an essential role of *SlLEA* genes in the regulation of these BP.

### Protein interaction network analysis

**Fig. 9:**
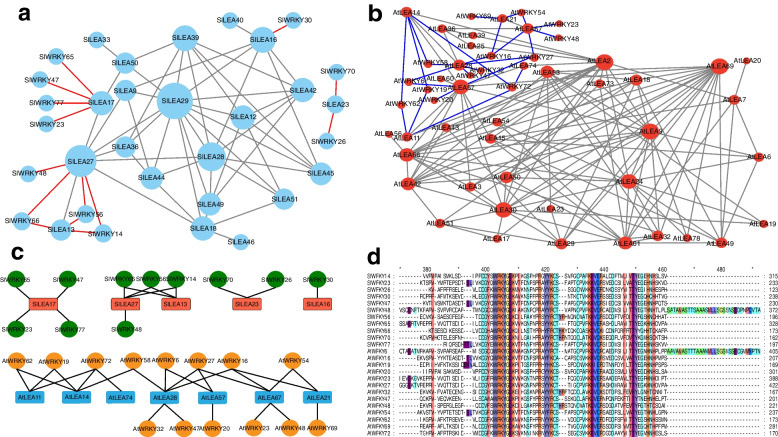
Protein interaction network diagrams of LEA and WRKY proteins in *S. lycopersicum* and *A. thaliana*. **a** Protein interaction network of SlLEA and SlWRKY proteins in *S. lycopersicum*. Network construction was performed using String 11.5. The basic settings were as follows: active interaction sources, experiments, databases, and gene fusion. The maximum number of interactors was 100, as calculated using the show simplification function to hide disconnected nodes in the network; the threshold for the confidence parameter was set to 0.40. Gray lines indicate interactions between different SlLEA proteins, and red lines indicate interactions between SlLEA and SlWRKY proteins. **b** Protein interaction network of AtLEA and AtWRKY proteins in *A. thaliana*. Gray lines indicate interactions among different AtLEA proteins, and blue lines indicate interactions between AtLEA and AtWRKY proteins. **c** Redrawing of a simpler and more intuitive protein interaction network diagram. The top panel shows the protein interaction network diagram of SlLEA and SlWRKY proteins. The bottom panel shows the protein interaction network diagram of AtLEA and AtWRKY proteins. **d** Comparative analysis of WRKY structural sequences interacting with LEA proteins in *S. lycopersicum* and *A. thaliana*

Based on String 11.5, we constructed the interaction network of SlLEA proteins with *S. lycopersicum* WRKY (SlWRKY) proteins in an attempt to improve our current understanding of the functional proteome-wide regulatory network (Fig. 9a). We found a total of five SlLEA proteins interacting with 11 SlWRKY proteins, including SlLEA16 with SlWRKY30, SlLEA23 with SlWRKY26/70, SlLEA13 with SlWRKY14/56/66, SlLEA27 with SlWRKY14/48/56/66, and SlLEA17 with SlWRKY23/47/65/77. They each covered different SlWRKY subclasses [50], of which Classes I and III each had one (i.e., SlWRKY14 and SlWRKY70, respectively) and Classes IIc and IId + IIe each had four (SlWRKY30/47/48/56) and five (SlWRKY23/26/65/66/77) SlWRKY members, respectively. Additionally, we constructed the interaction network of AtLEA proteins with AtWRKY proteins to further explore this network (Fig. 9b). We observed that a total of seven AtLEA proteins interacted with 14 AtWRKY proteins, including AtLEA11 with AtWRKY19/62/72, AtLEA14 with AtWRKY19/58/62/72, AtLEA74 with AtWRKY72, AtLEA28 with AtWRKY6/16/27/32/47/58, AtLEA57 with AtWRKY6/20/27, AtLEA67 with AtWRKY16/27/28/48/54, and AtLEA21 with AtWRKY16/54/69 (Fig. 9c). They also covered different AtWRKY subclasses [51], with three (AtWRKY20/32/58) and two (AtWRKY28/48) for Classes Ia and Ib, respectively; three (AtWRKY6/47/72) and two (AtWRKY27/69) for Classes IIa and IIb, respectively; and one (AtWRKY16) and two (AtWRKY54/62) for Classes IIe and III, respectively. In particular, AtWRKY19 is a member of the A1 subgroup of the family encoding MAPK/ERK kinases. In addition, we found that the core binding domains of SlWRKY and AtWRKY were highly conserved, suggesting that they may perform similar roles, such as interacting with LEA proteins (Fig. 9d).

### Changes in phenotypic and physiological indices in the *SlLEA6*-silenced lines under stress conditions

**Fig. 10:**
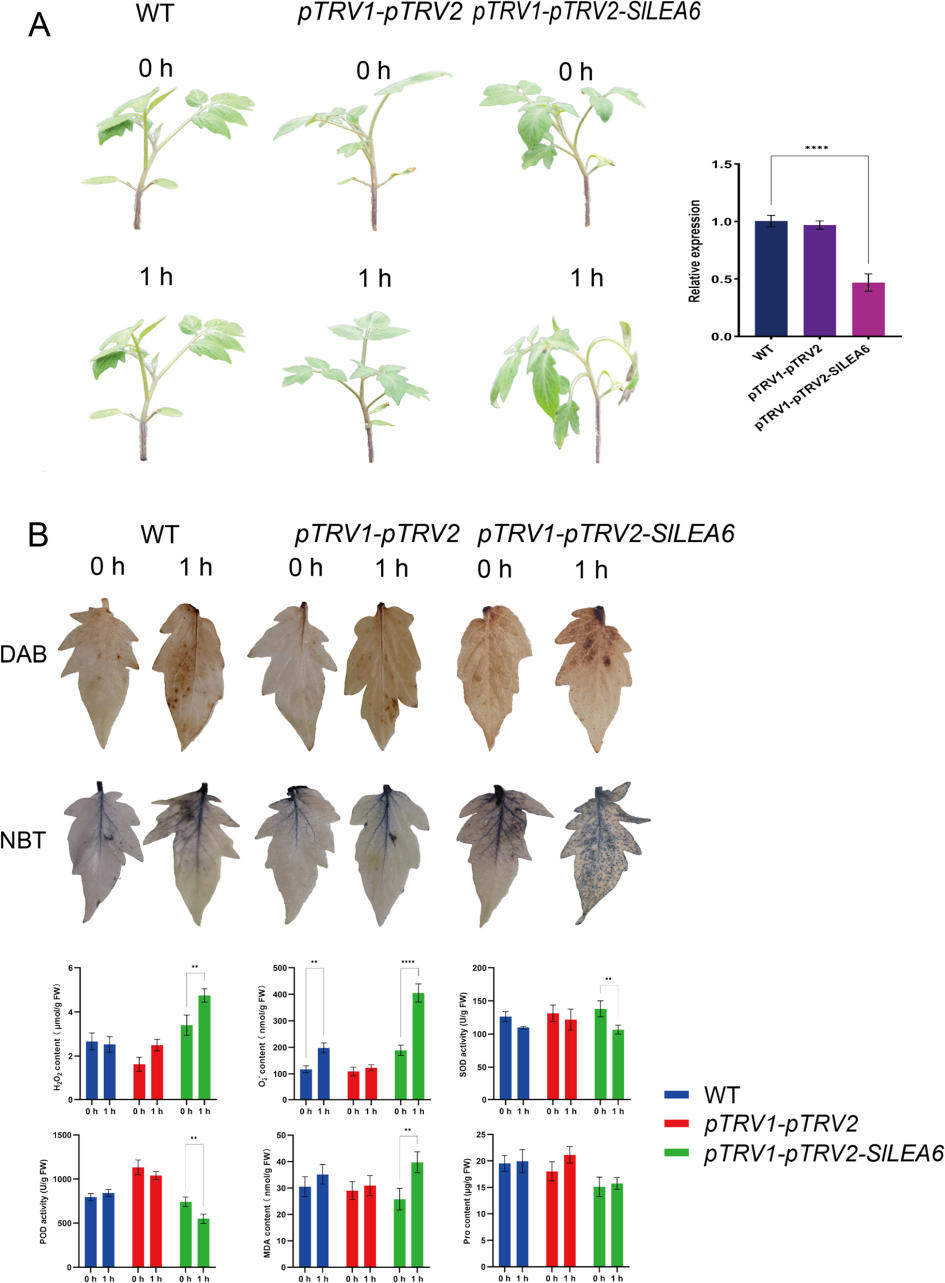
Phenotypic analysis and determination of physiological indices under drought stress conditions after *SlLEA6* silencing. **a** Phenotypic changes in plants after 1 h of drought stress and *SlLEA6* expression after being silenced. **b** DAB and NBT staining of leaves after 1 h of drought stress and determination of various physiological indices. Three independent biological replicates were included to calculate the mean. Error bars show the standard deviations (SD) of the three biological replicates. Values represent mean ± SD. Statistical significance of the differences was confirmed using Dunnett's multiple comparisons test (^*^ *P* < 0.05, ^**^ *P* < 0.01, ^***^ *P* < 0.001, and ^****^ *P* < 0.0001)

The VIGS technique is one of the most effective reverse genetics techniques used to characterize gene function. Our results of silencing and functional analyses of *SlLEA6* using the VIGS method showed that gene silencing of *SlLEA6* occurred at the posttranscriptional level in tomato leaves 2 weeks after injection inoculation, with a highly significant decrease in expression by approximately 60% compared to that in the negative control (Fig. 10a). After 1 h of salt, high, and low-temperature stress treatment respectively (note: *SlLEA6* expression peaked at this point), none of the plants showed any degree of phenotypic changes compared to those at 0 h (Additional file 15: Figure S12). Interestingly, after 1 h of drought stress, the *pTRV1-pTRV2-SlLEA6* plants exhibited more severe leaf curling and wilting than the negative and positive control plants compared to those at 0 h (Fig. 10a).

We further collected the top leaves of plants after drought stress for DAB and NBT staining and measured the enzymatic activities and physiological index contents of their biological antioxidant and ROS scavenging systems to systematically determine the effect of *SlLEA6* silencing on the drought tolerance of tomato plants (Fig. 10b). The results showed that after 1 h of drought stress, the superoxide anion (O_2_^−^), hydrogen peroxide (H_2_O_2_), malondialdehyde (MDA), and proline (Pro) contents were increased in the leaves of all plants compared to those at 0 h. However, none of the controls showed significant differences, except for the O_2_^−^ content of the negative control, indicating that the control treatment resulted in relatively less cell membrane and biomolecular damage. In contrast, the leaves of *pTRV1-pTRV2-SlLEA6* plants showed highly significant differences in the accumulation of O_2_^−^, H_2_O_2_, and MDA (but not of Pro), the contents of which were significantly higher than those in the negative and positive controls. Furthermore, superoxide dismutase (SOD) and peroxidase (POD) activities were highly significantly reduced, suggesting that the antioxidant and ROS defense systems in *pTRV1-pTRV2-SlLEA6* plants had a greater impact, which resulted in their inability to promptly catalyze degradation and increase oxidation, causing more severe cell damage. The above-mentioned phenotypic, physiological, and biochemical measurements suggested that silencing *SlLEA6* reduced the tolerance of tomato plants to drought stress and that it may be a candidate gene involved in drought stress response.

### Expression analysis of drought-induced *SlWRKY *genes in *SlLEA6*-silenced lines under drought stress

Given that *WRKY* TFs played a key role in drought stress tolerance, we further analyzed the expression pattern of drought-induced *SlWRKYs* under drought stress after constructing an interaction network between SlLEA and SlWRKY proteins to obtain information on *SlLEA6*-silenced strains involved in drought stress response. This analysis was conducted to explore their interaction and verify the potential regulatory functions. The selected drought-induced *SlWRKYs* were reported from previous studies [50] and consisted of eight genes, which were divided into two groups (upregulated and downregulated group), each containing the four genes with the strongest upregulation (*SlWRKY3*/*30*/*58*/*72*, corresponding to 13.27, 14.42, 125.37, and 36.76-fold changes, respectively) and downregulation (*SlWRKY33*/*39*/*45*/*46*, corresponding to -3.73, -3.25, -3.61, and -3.27-fold changes, respectively).

The expression patterns of drought-induced *SlWRKYs* were different in *SlLEA6-*silenced lines under drought stress (Additional file 16: Figure S13). Compared with that at 0 h, *SlWRKY3* in the upregulated group was not upregulated, but rather was downregulated, and there was no significant difference between the silenced lines and WT. *SlWRKY30* and *SlWRKY72* showed significant upregulation trends; however, there was no significant difference between the silenced line and WT. *SlWRKY58* showed a significant upregulation trend and there was a significant difference between the silenced lines and WT. Upregulation of *SlWRKY58* was significantly lower in the *SlLEA6-*silenced lines than in WT. Overexpression of *GhWRKY41* (i.e., the nearest homolog of *SlWRKY58*) in *Nicotiana benthamiana* has been shown to result in tolerance to drought and salt stress by enhancing stomatal closure and regulating ROS scavenging [52]. The antioxidant and ROS defense systems of *SlLEA6*-silenced lines were more negatively affected by ROS burst, implying that it positively regulated the expression of downstream drought-responsive genes to enhance drought tolerance in tomato plants during drought stress.

Compared with that at 0 h, *SlWRKY39* in the originally downregulated group was not downregulated, but rather was significantly upregulated; *SlWRKY33*/*46* were downregulated (but not significantly); and *SlWRKY45* was significantly downregulated. Their expression was not significantly different between the *SlLEA6-*silenced lines and WT. Under drought stress, only *SlWRKY45* expression was significantly reduced, suggesting that it may be a negative regulator of the response to drought stress in tomato plants; however, this requires further validation through functional analysis. Except for *SlWRKY3*/*39*, for which the expression patterns were the opposite to those originally reported [50], the expression patterns of the other *SlWRKYs* were largely consistent with the trends in the previously divided groups, indicating the reliability of the quantitative reverse transcription-polymerase chain reaction (qRT‒PCR) results.

## Discussion

The LEA family is widespread in plants and plays important roles, particularly in protecting cells from abiotic stressors and regulating normal growth and developmental processes [7, 28]. In the present study, we identified 60, 69, 65, and 60 *LEA* genes in the genomes of *S. lycopersicum*, *S. pimpinellifolium*, *S. pennellii*, and S. *lycopersicoides*, respectively. A total of 254 *LEA* genes were identified, which could be classified into eight clusters. We identified more genes and clusters than a previous study in which only 27 *LEA* genes were identified in the *S. lycopersicum* genome [14]. Continuous refinement of plant genome annotation and finer refined gene identification and classification are the most likely reasons for such differential results [19]. We compiled and summarized the main findings and analyzed them in comparison with previous results [14] to facilitate further clarification of the dynamics of the LEA family in tomatoes (Additional file 17: Table S4).

The phylogeny of LEA proteins in cultivated and wild tomato species was strongly correlated with gene structure, conserved structure domains, and motifs. These results not only support the reliability of phylogenetic tree analysis but also further support the evolutionarily conserved features of the LEA family. Similar to previous findings, the LEA_2 cluster was the largest subfamily among all eight clusters. This phenomenon was also corroborated by studies on *Sorghum bicolor* [53], *Camellia sinensis* [54], *Populus trichocarpa* [19], and *G. hirsutum* [25]. The LEA_6 cluster was the subfamily containing the smallest number of family members, which was also consistent with studies on *T. aestivum* [55], *V. vinifera* [16], and *G. hirsutum* [25]; i.e., it represents the smallest subfamily among most other plant species [56]. As expected, our results differed from those of studies on *O. sativa* [11] and *Brassica napus* [57] wherein the subfamilies with the largest number of LEA family members were dehydrin and LEA_4, respectively. This difference is because the composition of LEA subfamilies varies considerably among species.

LEA proteins of cultivated and wild tomato species exhibited a broad cytoarchitectural hierarchical distribution pattern and were mainly located in the nucleus (39.37%) and the protoplasmic membrane (26.77%). The findings of the present study were largely consistent with those of previous studies on *A. thaliana* [10] and *G. hirsutum* [25], which showed that LEA proteins are primarily situated in subcellular areas, with a positive correlation between subcellular positioning and the effect of LEA proteins in the cell.

Typically, genes that responded to stress included fewer introns than other genes, and members of the *LEA* family in cultivated and wild tomato species had relatively few introns, with 37.80% members belonging to the no-intron family. These findings are similar to those of previous studies into *LEA* families in other species [13, 19]. It may be that the *LEA* genes of cultivated and wild tomato species experienced a significant loss of introns during their evolution. Furthermore, the absence of introns reduces the deleterious effects on gene expression, i.e., it reduces the delay in transcript production and additional energy consumption, thus promoting rapid expression of *LEA* genes in cultivated and wild tomato species and improving adaptation to adversity [58–61].

Most LEA proteins in the same cluster shared one or more conserved structural domains and motifs, which indicated that they may play an important role in the primary and specific functions of the clade. Conserved structural domains are closely related to the physiological functions of proteins and are the structural basis of protein physiological functions. However, the structure varied greatly between the different branches, indicating that LEA proteins of cultivated and wild tomato species had functional complexity. Overall, it can be inferred with high probability from the distribution of conserved motifs that genes containing the same motifs are generated by the evolution of gene extensions within the same clade or taxon. Because different clusters consisted of different conserved motifs, it can also be preliminarily determined that they evolved from different ancestors with different conserved motifs [14].

In the present study, a phylogenetic tree analysis of LEA proteins among different model species was performed to elucidate the evolutionary relationships among the LEA families of cultivated and wild tomato species, *A. thaliana*, and *O. sativa*. The clustering of members into the same subfamily among different species implied that they have similar origins and evolutionary relationships. The different subfamilies contained LEA members from *O. sativa*, *A. thaliana*, and cultivated and wild tomato species, and their origins presumably appeared before the divergence of monocotyledons and dicotyledons [10, 11].

All *LEA* genes of cultivated and wild tomato species were distributed in a non-random pattern on their respective genomic chromosomes; they were more densely distributed on chromosomes 1 and 2, accounting for 32.68% of the total. In contrast, only *S. pimpinellifolium* had a single *LEA* gene distributed on chromosome 5. This layout makes it easier to associate that the genomes of cultivated and wild tomato species may have undergone multiple genome-wide replication events of chromosomal rearrangements and fusions during evolution, which allowed the expansion of numerous gene families [62, 63].

Universally, source drivers of gene family amplification mainly include segmental duplication, tandem duplication, and transposition events [64]. Our results showed that tandem duplication and segmental duplication events coexist in the *LEA* family and are the main mechanisms responsible for the increase in the number of *LEA*. Tandem duplication was much less frequent than segmental duplication, suggesting that the latter plays a major role in promoting *LEA* amplification. This mechanism of *LEA* amplification, wherein segmental duplication predominates, was similar to findings in *G. hirsutum* [25] and *P. trichocarpa* [19] and different from those in *A. thaliana* [10, 65] and *T. aestivum* [55]. In general, after a duplication event, a portion of duplicated genes is removed from the genome as a result of random mutations and loss of functions, and another portion is retained through functional differentiation [10]. This rapid expansion of the *LEA* family has adaptive implications for establishing desiccation tolerance [66]. The emergence of new genes is inseparable from the generation of replication events, and the diversity of gene functions requires the continuous replenishment of new genes, which can further help plants to resist adversity stress in the process of continuous evolution [59].

Usually, the AAs in proteins are considered to be under different selective pressures and are often measured by different *K*a/*K*s ratios, which can provide an important theoretical reference for species selection and evolution [67]. The *K*a/*K*s ratios of > 1, 1, and < 1 represent positive selection, neutral evolution, and purifying selection, respectively [68]. Our results indicated that the mean ratios of all segmental duplication and tandem duplication gene pairs were < 1, indicating that the *LEA* genes of cultivated and wild tomato species have experienced purifying selection through evolution, which may play an important role in sustaining the consistent stability of the biological structure of *LEA* genes over time. Because the *K*s value reflects the timing of homologous gene production to some extent, we used this value to make predictions regarding the dispersion time of *LEA* duplication events [69]. Based on the dispersion rate of 1.50 × 10^–8^ synonymous substitution rate loci per year, it can be deduced that segmental duplication of *LEA* genes occurred between 31.18 and 149.38 MYA and tandem duplication of *LEA* genes occurred between 3.17 and 92.64 MYA. It has been postulated that a minimum of two large-scale genome duplication events occurred during the evolution of the plant kingdom [69, 70]. This may imply that the expansion of *LEA* family members in cultivated and wild tomato species also contributed to these processes. Furthermore, we predicted that the tandem replication events of the *SpLEA55/SpLEA56*, *SlLEA53/SlLEA54*, and *SlydLEA50/SlydLEA51* gene pairs occurred at approximately 3.17, 3.33, and 3.94 MYA, respectively, i.e., after the tomato–*S. tuberosum* division [49]. Furthermore, similar synteny relationships existed between cultivated and wild tomato species for *LEA* genes. The majority of *LEA* genes formed approximately three synteny gene pairs, suggesting a conserved evolutionary role of the *LEA* family in cultivated and wild tomato species. Interestingly, more synteny relationships existed among *LEA* genes in monocotyledons than in dicotyleons. Some *LEA* genes in 10 species formed 2‒5 synteny gene pairs, which may have played an important role in the *LEA* family evolution.

Subsequently, by constructing synteny maps of *SlLEA* genes with the *LEA* genes of two monocotyledons (*Z. mays* and *O. sativa*) and two dicotyledons (*A. thaliana* and *S. tuberosum*), we further elucidated the sequence similarity of *SlLEA* genes with related *LEA* genes and homologous genes of other species. The synteny profiles of *SlLEA* genes were largely consistent with those of the remaining four species. We identified 14, 27, 47, and 57 repeat events in *Z. mays*, *O. sativa*, *A. thaliana*, and *S. tuberosum*, respectively. *SlLEA* genes had more synteny gene pairs with dicotyledons than with monocotyledons. In addition, *SlLEA* genes had no homologous genes in *Z. mays* and was the least homologous with *Z. mays*; had homologous genes in *O. sativa*, *A. thaliana*, and *S. tuberosum*; and was the most homologous with *S. tuberosum*. This was most likely because they belong to the same family (Solanaceae).

*Cis*-acting regulatory elements are required for gene regulation and expression processes. In particular, rapid and accurate identification of genes associated with specific functions in processes related to plant resistance to adversity and growth and development cannot be achieved without the aid of *cis*-acting regulatory element studies [71]. The *cis*-acting regulatory elements of *LEA* genes that were screened in the cultivated and wild tomato species were classified into four major categories—light response; abiotic stress environment or external stress response; phytohormone response; and *cis*-acting regulatory elements that participate in plant growth and development. In addition, some *LEA* genes also contained W-box motifs, which are combining locus for *WRKY* TFs. Among them, the first three major classes of *cis*-acting regulatory elements are widely available, and the promoter regions of all *LEA* genes contain several *cis*-acting regulatory elements from these three classes. This evidence suggested that members of the *LEA* family in cultivated and wild tomato species may be associated with multiple phytohormone responses and play key roles in abiotic stress defense as well as stress, wound response, and anaerobic induction. These inferences are consistent with the findings of previous studies on *cis*-acting regulatory elements of *LEA* family members in *P. trichocarpa* [19], *T. aestivum* [55], and *G. hirsutum* [25]. Of the *LEA* promoters in *A. thaliana*, 82% and 69% were reported to contain ABRE or LTRE *cis*-acting regulatory elements, respectively, in previous studies; among them, approximately 76.2% and 34.3% of *LEA* genes were significantly induced by ABA and low temperature, respectively [10]. In *Prunus mume*, 83.3% of the *LEA* promoters contained MYB *cis*-acting regulatory elements, of which approximately 63.3% of the *LEA* genes were induced by ABA [72]. These studies provide strong support for our inference. It is evident that the *LEA* genes of cultivated and wild tomato species play a critical role in stress resistance; these findings warrant further in-depth study.

Since gene function was visualized as changes in gene expression patterns, we first used normalized count-per-million (CPM) values to observe changes in the regulatory patterns of *LEA* genes in cultivated and wild tomato species under normal conditions. A differential regulation pattern was observed between them, and this up and downregulation may have been closely related to their abiotic stress tolerance-related expression patterns. Meanwhile, we analyzed the transcriptional expression profiles of *LEA* genes under salt, drought, and high-temperature stressors, which indicated the complexity and diversity of different gene regulation patterns in *LEA* genes in cultivated and wild tomato species. Thereafter, we examined the spatiotemporal expression differences among *SlLEA* genes in various tissues and during different developmental periods using transcriptional profiling data. *SlLEA* genes were expressed in multiple tissues, and expression levels varied greatly between tissues and periods. Overall, the expression of *SlLEA* genes was generally low in meristematic tissues, leaves, and flowers and generally high in seeds and fruits; this represented a tissue-specific expression profile that implies their involvement in specific tissue and developmental processes. Our results are supported by studies into the role of *LEA* genes in protecting *Citrus unshiu* [73], *S. tuberosum* [74], *Cucumis melo*, and *Citrillus lanatus* [75] against abiotic stressors while also functioning in normal cellular functions and fruit developmental stages. We also observed that certain *SlLEA* genes exhibited unique expression profiles and that their persistent high expression in specific organs may play a key role in the growth and development of tomato plants, in particular in regulating seed maturation and fruit maturation from immaturity to ripening. This finding was similar to the results of previous findings that showed that LEA proteins accumulate at high levels during late seed maturation and are absent after germination [55, 76]

Moreover, we also investigated the spatiotemporal expression modes of *SlLEA* genes in response to various stressors and phytohormone treatments using qRT‒PCR in an attempt to elucidate the possible effects of various *SlLEA* family members. *SlLEA* expression patterns were affected by a wide range of abiotic stressors and phytohormone treatments, and certain *SlLEA* genes were rapidly induced and persistently highly expressed during specific periods, which demonstrated that they performed particular functions under different stressors and hormone treatments. Overall, *SlLEA* expression was more sensitive to high salt and drought stressors than extreme temperature. We also observed that some *SlLEA* genes showed similar expression patterns in response to various abiotic stressors and phytohormone treatments. *SlLEA6*/*27* may be the key genes in the *SlLEA* family involved in defense and stress response to external stressors. *TaLEA3-2*/*6–3* were previously reported to be strongly responsive to drought and salt stress in *T. aestivum* [55]. *ZmLEA31* gene in *Z. mays* was upregulated under drought, high-salt, low-temperature, and IAA treatments [12], which was similar to our findings. Furthermore, overexpression of *LEA* genes resulted in enhanced salt tolerance, drought tolerance, sensitivity to ABA, osmotic stress tolerance, and oxidative stress in transgenic *A. thaliana* [35, 36], *O. sativa* [37], and *N. tabacum* [38]. In the present study, we found that silencing *SlLEA6* significantly increased the H_2_O_2_, O_2_^−^, and MDA contents in leaves while significantly reducing SOD and POD activities. This caused a greater impact on biological antioxidant and ROS defense systems, increased oxidation (thereby increasing the degree of cellular damage), and led to an increase in leaf wilting, reducing the tolerance of tomato plants to drought stress. The *SlLEA6* identified using VIGS technology can be used as a candidate gene for subsequent studies to improve the current understanding of drought tolerance in tomato plants.

We performed GO annotation analysis of 254 *LEA* genes in cultivated and wild tomatoes and 60 *SlLEA* genes to obtain the BP, MF, and CC of all *LEA* genes. A series of BP related to *SlLEA* genes were clarified and were mostly predicted to function in response to desiccation and water deficit as well as to abiotic stimuli and stressful environments. The enrichment results also revealed that the most abundant functions in their CC involved the membrane, cellular, and cytoplasmic fractions. This was similar to the analysis of the *LEA* family in *G. hirsutum* and is presumably consistent with their function in securing membranes and enzymes to maintain cellular activity under drought stress [25, 32].

*WRKY* TFs are one of the most populated families of transcriptional regulators that regulate plant growth and development and can interact with all kinds of proteins or TFs to perform their corresponding biological functions and regulate plant defense responses. WRKY proteins can bind to the W-box in the promoter region of target genes to activate or inhibit the expression of downstream genes, thereby regulating their stress response [77]. However, the functional mechanisms underlying the interaction between SlLEA and SlWRKY proteins remain poorly understood. The protein interactions regulatory network constructed in this study showed that *S. lycopersicum* and *A. thaliana* LEA proteins interacted with multiple WRKY proteins to regulate a variety of physiological processes. Our analysis of the expression of drought-induced *SlWRKY* genes in *SlLEA6*-silenced lines under drought stress showed that the expression patterns of the eight selected *SlWRKYs* differed. Only *SlWRKY45* expression was significantly reduced under drought stress, indicating that it may be a negative regulator of tomato plant response to drought stress; however, this requires further verification via functional analysis. In addition, the increased expression of *SlWRKY58* was significantly lowered in *SlLEA6-*silenced lines compared to that in WT; overexpression of *GhWRKY41* (the nearest homolog of *SlWRKY58*) in *N. benthamiana* has been shown to confer drought and salt-stress tolerance by enhancing stomatal closure and regulating ROS clearance [52]. Contrastingly, the *SlLEA6*-silenced lines did have a serious negative impact on antioxidant and ROS defense systems, which implied that they positively regulate the expression of downstream drought-responsive genes under drought stress to enhance drought resistance in tomato plants.

To date, AtWRKY19 [78], AtWRKY22/29 [79], AtWRKY38/48/62 [80], and AtWRKY72 [81] in *A. thaliana* have been reported to be involved in biotic stress defense; AtWRKY25/26/33 [82] and AtWRKY39 [83] respond to high-temperature stress; AtWRKY34 [84] responds to low-temperature stress; AtWRKY25/33 [82], AtWRKY46 [85], and AtWRKY6/8/22/30/39/48/53/75 [86] respond to salt, drought, and oxidative stress; AtWRKY53/54/70 [87] and AtWRKY57 [88] are involved in regulating leaf senescence mechanisms, and AtWRKY27 [89] plays a key role in pollen growth and development. In *S. lycopersicum*, SlWRKY1/11 [90], SlWRKY39 [91], and SlWRKY70 [92] are involved in biotic stress defense; SlWRKY12/13/23/50/51 [93] respond to low-temperature stress; SlWRKY39 [91], SlWRKY23/31/32/33/41/57/74 [94], SlWRKY58 [50], and SlWRKY81 [95] respond to salt and drought stress; and SlWRKY23 [96] and SlWRKY16/17/53/54 [97] play key roles in growth and fruit ripening. Meanwhile, SlWRKY39/40/43/45/46 are closely homologous to AtWRKY18/40/60 in Class IIa and have been shown to respond to both abiotic and biotic stressors [98]. These previous findings on AtWRKY and SlWRKY proteins imply that some of the AtLEA and SlLEA proteins identified in the present study that interact with WRKY proteins may also have corresponding biological functions. Nonetheless, it should be emphasized that slight variations in the DNA binding domain may have critical implications for binding specificity and that sequence homologs may be very similar but have various functions [99].

## Conclusions

In summary, the present study provides a comprehensive and systematic study of the LEA family in cultivated and wild tomato species. We identified 254 *LEA* genes divided into eight clusters with different physicochemical properties. They were non-randomly distributed on chromosomes, and promoter regions contained more *cis*-acting regulatory elements linked to abiotic stress tolerance and phytohormone response. Evolutionary analysis revealed replication events, purifying selection, divergence time, intraspecific and interspecific homology, and synteny of *LEA* genes. Transcriptional profiling and gene expression pattern analyses indicated specific roles of certain *SlLEA* genes during growth and development or exposure to various abiotic stressors and phytohormone treatments. Gene functional annotations were then developed and protein-based interaction networks were constructed to further elucidate the gene and functional proteomic regulatory networks. Finally, *SlLEA6* was shown to be a candidate gene involved in response to drought stress. Our results provide strong support for an improved appreciation of the evolutionary history and function of *LEA* genes and lay the foundation for subsequent in-depth studies of the molecular and biological functions of this family.

## Materials and methods

### LEA identification and characterization

Genome-wide data were obtained from the Sol Genomics Network (SGN) database (https://solgenomics.net/), and based on a hidden Markov model (HMM), the conserved domains of LEA proteins were obtained. The potential HMM profile was utilized as an inquiry separately against the genomes of cultivated (*S. lycopersicum*) and wild tomato species (*S. pimpinellifolium*, *S. pennellii*, and *S. lycopersicoides*) via the HMMER search (https://www.ebi.ac.uk/Tools/hmmer/) [100]. Subsequently, the clustering comparison was first performed and all redundant sequences were discarded. The Simple Modular Architecture Research Tool (SMART) database (http://smart.embl-heidelberg.de/) [101] and Pfam database (http://pfam.xfam.org/) [102] were then used to confirm the existence of LEA domains. Physicochemical parameters such as MW and pI of LEA proteins were anticipated using the ExPASy server tool (http://www.web.expasy.org/protparam/). The subCELlular LOcalization predictor (CELLO) website (http://cello.life.nctu.edu.tw/) was used for analyzing the subcellular location of all LEA proteins.

### Phylogeny, gene structure, conserved domains, and motif analyses of LEA proteins

LEA protein sequences were clustered to construct an evolutionary tree, and the coding regions and genomic sequences were compared to analyze the gene structure. The conserved structural domains and motifs of proteins were evaluated using the HMMER search and Multiple Expectation Maximization for Motif Elicitation (MEME) website. (https://meme-suite.org/meme/tools/meme) Finally, all of these were visualized using TBtools [103].

### Phylogenetic tree analysis of LEA proteins

To investigate the phylogenetic relationships between the model crops (*A. thaliana* and *O. sativa*) and the LEA family, we first obtained the protein sequences of AtLEA and OsLEA after screening and identification using the HMMER search. Then, a phylogenetic tree analysis was performed using MEGA 11 software [104] and the maximum likelihood (ML) method with bootstrap analysis for 1000 repetitions.

### Chromosome distribution, replication events, purification pressure, and synteny analysis

The DNA sequences of each member of the *LEA* family were localized to the genomes of cultivated and wild tomato species, and the gene distribution on chromosomes was calculated and mapped using TBtools. Gene duplication events for *LEA* genes were calculated using the MCScanX toolkit [105]. We used the latest KaKs_Calculator 3.0 software to calculate the *K*a and *K*s ratios for the duplicate gene pairs of *LEA* [106]. Then, the replication date of each gene pair was calculated using the *K*s value by applying the formula T = Ks/2λ, where λ was assumed to be 1.5 × 10^–8^ synonymous/substitution site/year [69]. Single-copy homologous genes were obtained using diamond comparison in the OrthoFinder 2.5.2 software [107], evolutionary divergence trees of different species were constructed using the RAxML 8.0 tool [108], and fossil times were added to the evolutionary trees using the TimeTree database (http://www.timetree.org/). Then, the mcmctree command in PAML 4.9 software [109] was used to estimate the divergence times of different species. Moreover, the synteny of *LEA* genes between different species was calculated, analyzed, and visualized using the dual synteny plotter module in TBtools.

### Analysis of the *cis*-acting regulatory elements of *LEA* genes

Sequences in the range of 2000 base pairs (bp) upstream of the start codon (ATG) in *LEA* genes were downloaded from the genomes of cultivated and wild tomato species and analyzed using the PlantCARE database (http://bioinformatics.psb.ugent.be/webtools/plantcate/html) for promoter *cis*-acting regulatory element screening.

### Analysis of *LEA* expression pattern

The CPM values for *LEA* genes of *S. lycopersicum* and its wild relatives (*S. pimpinellifolium*, *S. habrochaites*, and *S. pennellii*) were obtained from the Maloof Lab database (http://malooflab.phytonetworks.org/apps/tomato-expression/). Transcriptomic data of cultivated and wild tomato species under stress conditions were downloaded from the Gene Expression Omnibus (GEO) database (https://www.ncbi.nlm.nih.gov/geo/). Salt stress corresponds to the GEO accession GSE16401 [110], and drought and high-temperature stressors correspond to the GEO accession GSE22304 [111]. *SlLEA* expression data in various tissues (including seeds, roots, meristem, leaves, flowers, and fruits) are available on the TomExpress database, i.e., a unified tomato RNA-Seq platform (http://tomexpress.toulouse.inra.fr/). Cluster heat map analysis was conducted using the OmicShare tool, i.e., a free online data analysis platform (https://www.omicshare.com/tools).

### Plant material and treatments

*S. lycopersicum* var. M82 was used as experimental material. Seeds were cultivated in a 3:1 (w/w) mixture of nutrient soil and vermiculite, and plants were grown in an artificial greenhouse at a controlled temperature of 25 ± 1 ℃, relative humidity of 50 ± 10%, and a 16 h/8 h photoperiod (daytime: 08:00‒00:00). After 4 weeks, seedlings were sprayed with 100 µM ABA, 100 µM MeJA, 50 µM *rac*-GR24, and 100 µM GABA for analysis of the response to phytohormone treatment. Seedlings were sprayed with double distilled water (ddH_2_O) in the control treatment. Each solution was sprayed evenly on each leaf, and spraying was stopped before the solution dripped from the leaf. Similarly, for 4-week-old seedlings, abiotic stress responses were analyzed after treatment with 200 mM NaCl and 300 mM mannitol. The corresponding solution was slowly poured from the top of the seedlings into the planting trays with holes at the bottom, and watering was ceased when the solution began to leak from the holes, indicating saturation. Seedling were placed in 4 ℃ and 35 ℃ light-temperature incubators for low and high temperature treatments, respectively, and the control group followed the standard light and temperature as described previously. The control and treated seedlings were collected at 0, 1, 3, 6, 12, and 24 h after processing, whereby the top first fully expanded leaves of three uniformly grown seedlings of each treatment were mixed as biological replicates and replicated three times. Samples were frozen in liquid nitrogen and stockpiled in an ultra-low temperature refrigerator at -80 ℃ until use.

### RNA extraction, qRT‒PCR, and statistical analysis

Total RNA was extracted from the harvested samples using the Polysaccharide Polyphenol Plant Total RNA Extraction Kit (DP441, Tiangen, Beijing, China) according to the manufacturer's instructions. First-strand cDNA synthesis was completed using the 5 × All-In-One RT MasterMix (with AccuRT Genomic DNA Removal Kit) (G492, ABM, Vancouver, Canada) according to the manufacturer's instructions. Primers specific for *SlLEA* and *SlWRKY* genes were designed using the NCBI primer design tool (https://www.ncbi.nlm.nih.gov/tools/primer-blast/index.cgi?LINK_LOC=BlastHome), and *Actin* (*Solyc03g078400.2*) was used as an internal reference control. qRT‒PCR was performed on a LightCycler® real-time fluorescent quantitative PCR system (Roche, Basel, Switzerland) using ChamQ Universal SYBR qPCR Master Mix (Q711, Vazyme, Nanjing, China) and the primers listed in Additional file 18: Table S5. The amplification process consisted of one cycle of 95 °C pre-denaturation for 30 s, followed by 40 cycles of 95 °C denaturation for 5 s, and 60 °C annealing and extension for 30 s. Finally, one cycle of 95 °C for 15 s, 60 °C for 60 s, and 95 °C for 15 s was performed for melting curve acquisition. For each sample, three technical replicates were performed to calculate the mean cycle threshold (C_t_) value. Relative expression was calculated using the 2^−ΔΔCt^ method [112]. All data were subjected to variance analysis; the mean of values from all treatments is presented. Three independent biological replicates were used for each determination. A one-way analysis of variance (ANOVA) was performed using GraphPad Prism for Windows (version 9.0.0, GraphPad Software, San Diego, CA, USA), followed by Dunnett's multiple comparisons test.

### GO and protein interaction network analyses

GO analysis of *LEA* genes in cultivated and wild tomato species was performed using the OmicShare tool. The ensemble IDs of *LEA* genes were loaded into the program and used to complete the three steps of functional analysis—(i) mapping to each term of the GO database (http://www.geneontology.org/) and calculating the number of genes per term; (ii) applying hypergeometric tests to recognize GO entries that were remarkably enriched in genes contrasted to the total genomic context; and (iii) using a corrected-*P* value ≤ 0.05 as the threshold. GO terms that satisfied this requirement were recognized as being significantly enriched in genes. Thus, the MF, CC, and BP involved in the genes were described separately.

The protein sequence of SlWRKY was acquired from the *S. lycopersicum* genome database and localized to the AtWRKY protein on The *Arabidopsis* Information Resource (TAIR) website (https://www.arabidopsis.org/) using the BLASTP tool. Subsequently, the interactions of SlLEA and SlWRKY proteins and of AtLEA and AtWRKY proteins were predicted according to String 11.5, and their interaction networks were plotted. Sequence alignment of SlWRKY and AtWRKY proteins was performed using MUSCLE 5.1 software.

### VIGS vector construction, gene silencing, and determination of physiological indices

VIGS vectors for the target genes were designed and constructed using the SGN VIGS tool (https://vigs.solgenomics.net/), following the procedure reported in the literature [113]. Briefly, *pTRV1*, *pTRV2* empty, and *pTRV2* recombinant vectors were cloned into *Agrobacterium tumefaciens* GV3101 (pSoup-p19), cultured overnight in a shaker, centrifuged to collect the organisms, and adjusted with a resuspension solution to a final concentration corresponding to OD_600_ = 1.0. The *pTRV1-pTRV2* empty vector and *pTRV1-pTRV2* recombinant vector resuspension were mixed at a ratio of 1:1 (v/v) and incubated at 28 °C for 3 h in darkness and then infiltrated into the abaxial surface of the leaves of 4-week-old tomato plants using a 1-mL syringe without the needle. After 12 h of dark treatment, a normal photoperiod was provided. Untreated plants served as the negative controls and plants inoculated with *pTRV1-pTRV2* empty served as the positive controls. Abiotic stress conditions were applied 2 weeks later as previously described. Top leaves were collected from the same parts at 0 h and 1 h post-stress and stained using an NBT solution (0.1%, pH 7.8) (SL18061, Coolaber, Beijing, China) and a DAB solution (1 mg/mL, pH 3.8) (SL1805, Coolaber, Beijing, China), respectively. Six test kits (SA-1-G, H2O2-1-Y, MDA-1-Y, PRO-1-Y, SOD-1-Y, and POD-1-Y, Comin, Suzhou, China) were used sequentially to detect O_2_^−^, H_2_O_2_, MDA, Pro, SOD, and POD activity/content according to the manufacturer's instructions; three replicates were included.

## Supplementary Information

**Additional file 1:** **Table S1.** Identification and characterization of LEAs in cultivated and wild tomato species.

**Additional file 2:** **Table S2.** Basic information on the AtLEAs and OsLEAs re-predicted, identified, and renamed in this study.

**Additional file 3:** **Table S3. **The list of replication events,* Ka*/*Ks* ratios, and their replication time for *LEAs*.

**Additional file 4:** **Figure S1.** Expression patterns of *SlLEAs* under normal conditions (control). Three independent biological replicates were included to calculate the mean. Error bars show the SD of the three biological replicates. Values represent mean ± SD. Statistical significance of the differences was confirmed using Dunnett's multiple comparisons test (^*^P<0.05, ^**^P<0.01, ^***^P<0.001, and ^****^P<0.0001).

**Additional file 5:** **Figure S2.** Expression patterns of *SlLEAs* under simulated drought stress (300 mM mannitol). Three independent biological replicates were included to calculate the mean. Error bars show the SD of the three biological replicates. Values represent mean ± SD. Statistical significance of the differences was confirmed using Dunnett's multiple comparisons test (^*^P<0.05, ^**^P<0.01, ^***^P<0.001, and ^****^P<0.0001).

**Additional file 6:** **Figure S3.** Expression patterns of *SlLEAs* under simulated high salt stress (200 mM NaCl). Three independent biological replicates were included to calculate the mean. Error bars show the SD of the three biological replicates. Values represent mean ± SD. Statistical significance of the differences was confirmed using Dunnett's multiple comparisons test (^*^P<0.05, ^**^P<0.01, ^***^P<0.001, and ^****^P<0.0001).

**Additional file 7:** **Figure S4.** Expression patterns of *SlLEAs* under simulated high-temperature stress (35°C). Three independent biological replicates were included to calculate the mean. Error bars show the SD of the three biological replicates. Values represent mean ± SD. Statistical significance of the differences was confirmed using Dunnett's multiple comparisons test (^*^P<0.05, ^**^P<0.01, ^***^P<0.001, and ^****^P<0.0001).

**Additional file 8:** **Figure S5.** Expression patterns of *SlLEAs* under simulated low-temperature stress (4°C). Three independent biological replicates were included to calculate the mean. Error bars show the SD of the three biological replicates. Values represent mean ± SD. Statistical significance of the differences was confirmed using Dunnett's multiple comparisons test (^*^P<0.05, ^**^P<0.01, ^***^P<0.001, and ^****^P<0.0001).

**Additional file 9:** **Figure S6.** Expression patterns of *SlLEAs* in response to ddH_2_O control treatment. Three independent biological replicates were included to calculate the mean. Error bars show the SD of the three biological replicates. Values represent mean ± SD. Statistical significance of the differences was confirmed using Dunnett's multiple comparisons test (^*^P<0.05, ^**^P<0.01, ^***^P<0.001, and ^****^P<0.0001).

**Additional file 10:** **Figure S7.** Expression patterns of *SlLEAs* in response to 100 µM ABA treatment. Three independent biological replicates were included to calculate the mean. Error bars show the SD of the three biological replicates. Values represent mean ± SD. Statistical significance of the differences was confirmed using Dunnett's multiple comparisons test (^*^P<0.05, ^**^P<0.01, ^***^P<0.001, and ^****^P<0.0001).

**Additional file 11:** **Figure S8.** Expression patterns of *SlLEAs* in response to 100 µM MeJA treatment. Three independent biological replicates were included to calculate the mean. Error bars show the SD of the three biological replicates. Values represent mean ± SD. Statistical significance of the differences was confirmed using Dunnett's multiple comparisons test (^*^P<0.05, ^**^P<0.01, ^***^P<0.001, and ^****^P<0.0001).

**Additional file 12:** **Figure S9.** Expression patterns of *SlLEAs* in response to 50 µM *rac*-GR24 treatment. Three independent biological replicates were included to calculate the mean. Error bars show the SD of the three biological replicates. Values represent mean ± SD. Statistical significance of the differences was confirmed using Dunnett's multiple comparisons test (^*^P<0.05, ^**^P<0.01, ^***^P<0.001, and ^****^P<0.0001).

**Additional file 13:** **Figure S10.** Expression patterns of *SlLEAs* in response to 100 µM GABA treatment. Three independent biological replicates were included to calculate the mean. Error bars show the SD of the three biological replicates. Values represent mean ± SD. Statistical significance of the differences was confirmed using Dunnett's multiple comparisons test (^*^P<0.05, ^**^P<0.01, ^***^P<0.001, and ^****^P<0.0001).

**Additional file 14:** **Figure S11.** GO analysis of 254 LEAs in this study.

**Additional file 15:** **Figure S12.** Phenotypic analysis under abiotic stress conditions after silencing of *SlLEA6* (**a**: salt stress; **b**: high temperature stress; **c**: low temperature stress).

**Additional file 16:** **Figure S13. **Expression patterns of eight drought-induced *SlWRKYs* in *SlLEA6* silenced lines under drought stress. Three independent biological replicates were included to calculate the mean. Error bars show the SD of the three biological replicates. Values represent mean ± SD. Statistical significance of the differences was confirmed using Dunnett's multiple comparisons test (^*^P<0.05, ^**^P<0.01, ^***^P<0.001, and ^****^P<0.0001).

**Additional file 17:** **Table S4.** Comparison and summary of the main study results.

**Additional file 18:** **Table S5.** The primer sequences used in this study for qRT-PCR.

## Data Availability

All data generated or analyzed during this study are included in this published article and its supplementary information file. Transcriptome sequencing data used during the study were obtained from Maloof Lab (http://malooflab.phytonetworks.org/apps/tomato-expression/), TomExpress (http://tomexpress.toulouse.inra.fr/), and GEO databases (GEO accession GSE16401 and GSE22304), respectively.

## Abbreviations

LEA: Late embryogenesis abundant
Pfam: The protein families
IDs: Identity documents
ROS: Reactive oxygen species
TFs: Transcription factors
AA: Amino acids
ABA: Abscisic acid
GO: Gene ontology
VIGS: Virus-induced gene silencing
SMP: Seed maturation protein
MW: Molecular weight
pI: Isoelectric point
GRAVY: Grand average of hydropathy index
UTR: Untranslated regions
*K*a: Non-synonymous substitution
*K*s: Synonymous substitution
MYA: Million years ago
MeJA: Methyl jasmonate
GA: Gibberellins
IAA: Auxin
GABA: γ-Aminobutyric acid
BP: Biological processes
MF: Molecular functions
CC: Cellular components
MEKK: MAPK/ERK kinases
O_2_: Superoxide anion
H_2_O_2_: Hydrogen peroxide
MDA: Malondialdehyde
Pro: Proline
SOD: Superoxide dismutase
POD: Peroxidase
CPM: Count-per-million
SGN: Sol Genomics Network
HMM: Hidden Markov model
SMART: Simple modular architecture research tool
MEME: Maximization for motif elicitation
ML: Maximum likelihood
GEO: Gene expression omnibus
ddH_2_O: Distilled water
qRT-PCR: Quantitative real-time PCR
C_t_: Cycle threshold
TAIR: The *Arabidopsis* information resource
