# Correction: The LEA gene family in tomato and its wild relatives: genome-wide identifcation, structural characterization, expression profling, and role of *SlLEA6* in drought stress

**DOI:** 10.1186/s12870-023-04052-x

**Published:** 2023-01-26

**Authors:** Chunping Jia, Bin Guo, Baike Wang, Xin Li, Tao Yang, Ning Li, Juan Wang, Qinghui Yu

**Affiliations:** 1 Institute of Horticulture Crops, Xinjiang Academy of Agricultural Sciences (Key Laboratory of Genome Research and Genetic Improvement of Xinjiang Characteristic Fruits and Vegetables), Urumqi, China; 2 College of Life Science and Technology, Xinjiang University, Urumqi, China; 3 College of Computer and Information Engineering, Xinjiang Agricultural University, Urumqi, China

**Correction:***BMC Plant Biol***22, 596 (2022)**

**https://doi.org/10.1186/s12870-022-03953-7**

Following the publication of the original article [1], the authors identified an error in the affiliation numbers of some of the authors. The correct authors with their corresponding affiliation numbers are presented below:

- Chunping Jia^1,2†^, Bin Guo^1,3†^, Baike Wang^1^ , Xin Li^1,3^, Tao Yang^1^, Ning Li^1^ , Juan Wang^1*^ and Qinghui Yu^1,2*^

The correction does not have any effect on the results or conclusions of the paper. The original article [1] has been corrected.
