# Supplementary figures and images for: The LEA gene family in tomato and its wild relatives: genome-wide identification, structural characterization, expression profiling, and role of SlLEA6 in drought stress
Source: BMC Plant Biol. 2022 Dec 19;22:596. doi: 10.1186/s12870-022-03953-7 (PMC9762057; doi:10.1186/s12870-022-03953-7)

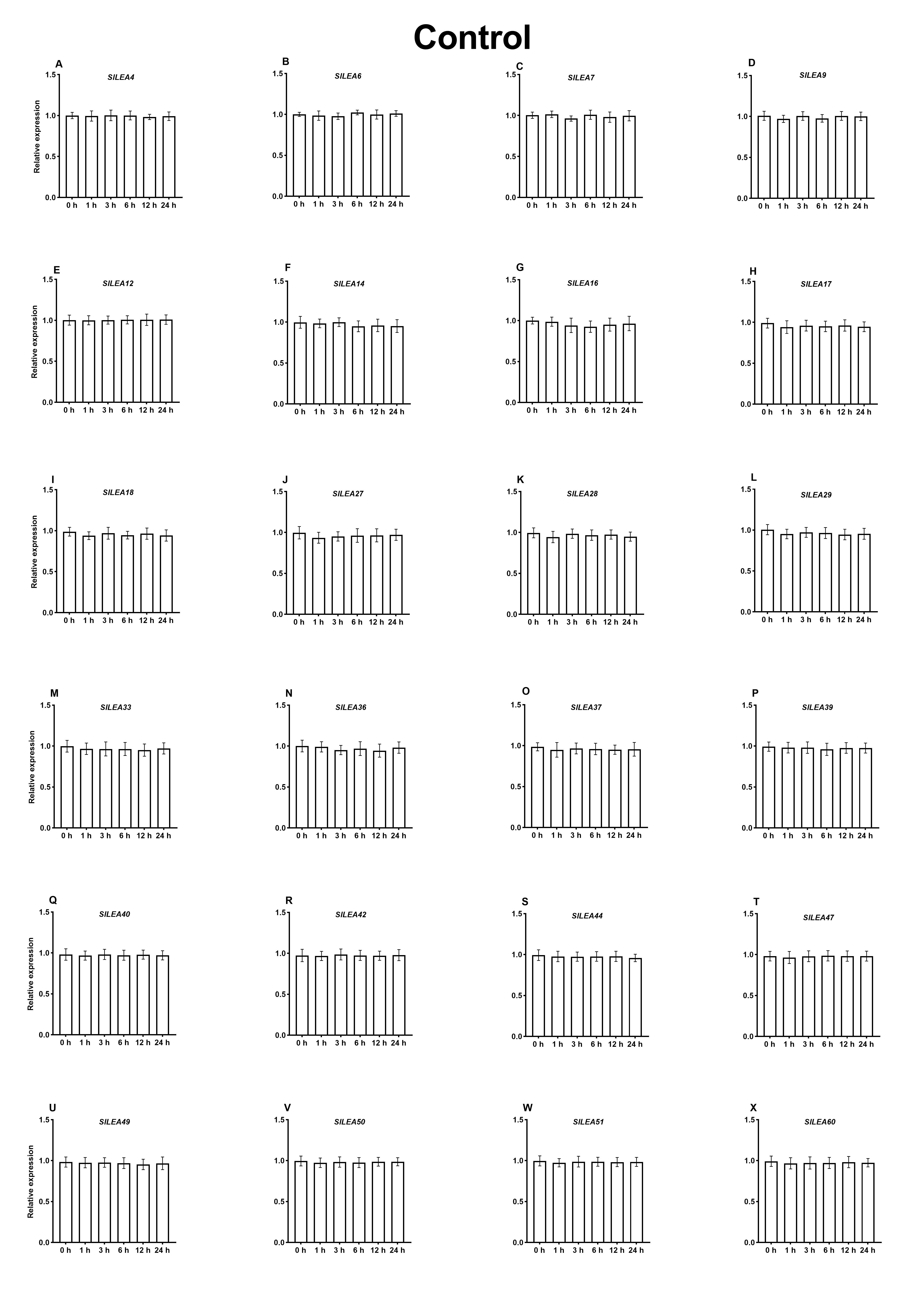

Supplement: Supplementary file 4 — Additional file 4: Figure S1. Expression patterns of SlLEAs under normal conditions (control). Three independent biological replicates were included to calculate the mean. Error bars show the SD of the three biological replicates. Values represent mean ± SD. Statistical significance of the differences was confirmed using Dunnett's multiple comparisons test (*P<0.05, **P<0.01, ***P<0.001, and ****P<0.0001). [file 12870_2022_3953_MOESM4_ESM.tif]

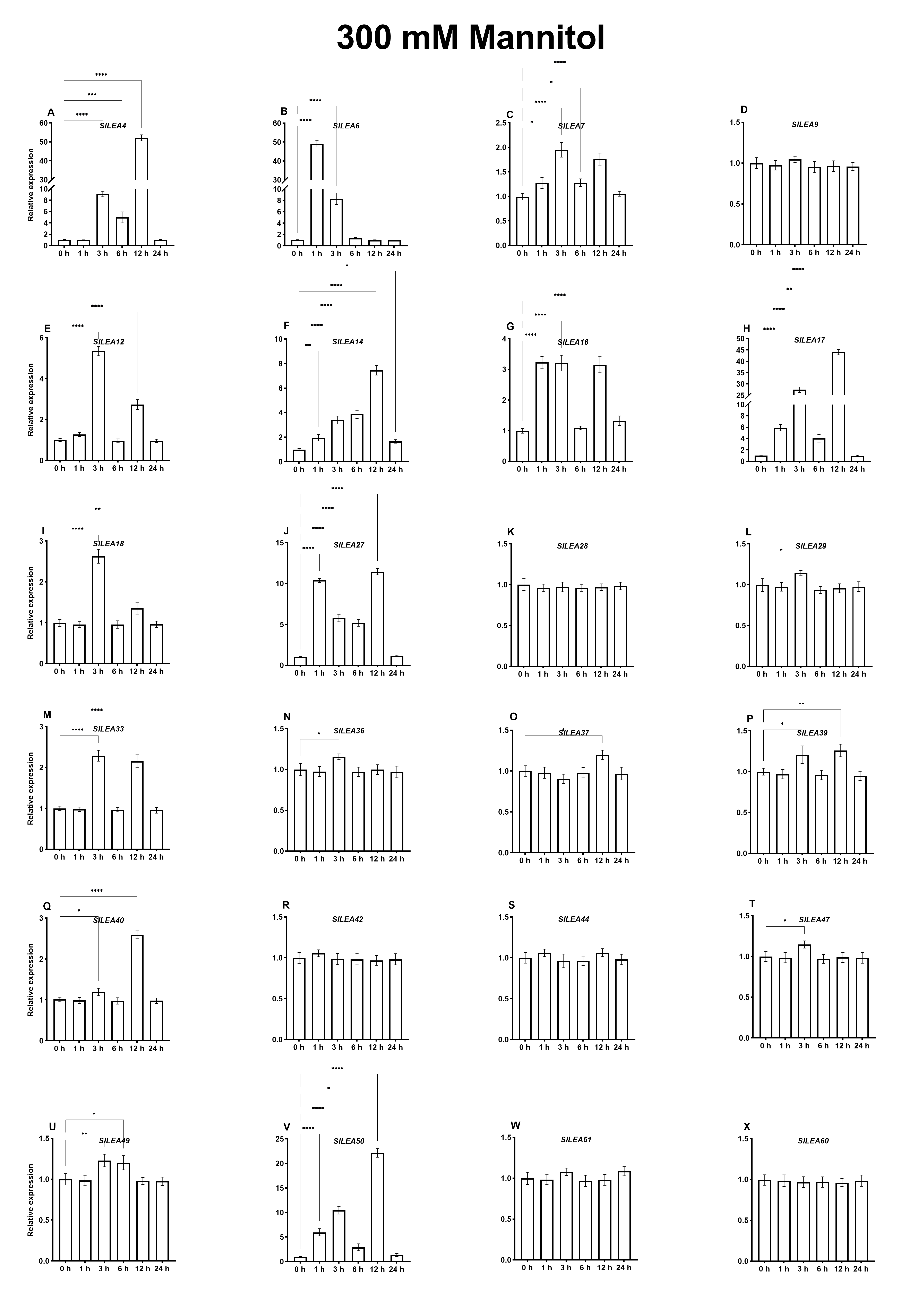

Supplement: Supplementary file 5 — Additional file 5: Figure S2. Expression patterns of SlLEAs under simulated drought stress (300 mM mannitol). Three independent biological replicates were included to calculate the mean. Error bars show the SD of the three biological replicates. Values represent mean ± SD. Statistical significance of the differences was confirmed using Dunnett's multiple comparisons test (*P<0.05, **P<0.01, ***P<0.001, and ****P<0.0001). [file 12870_2022_3953_MOESM5_ESM.tif]

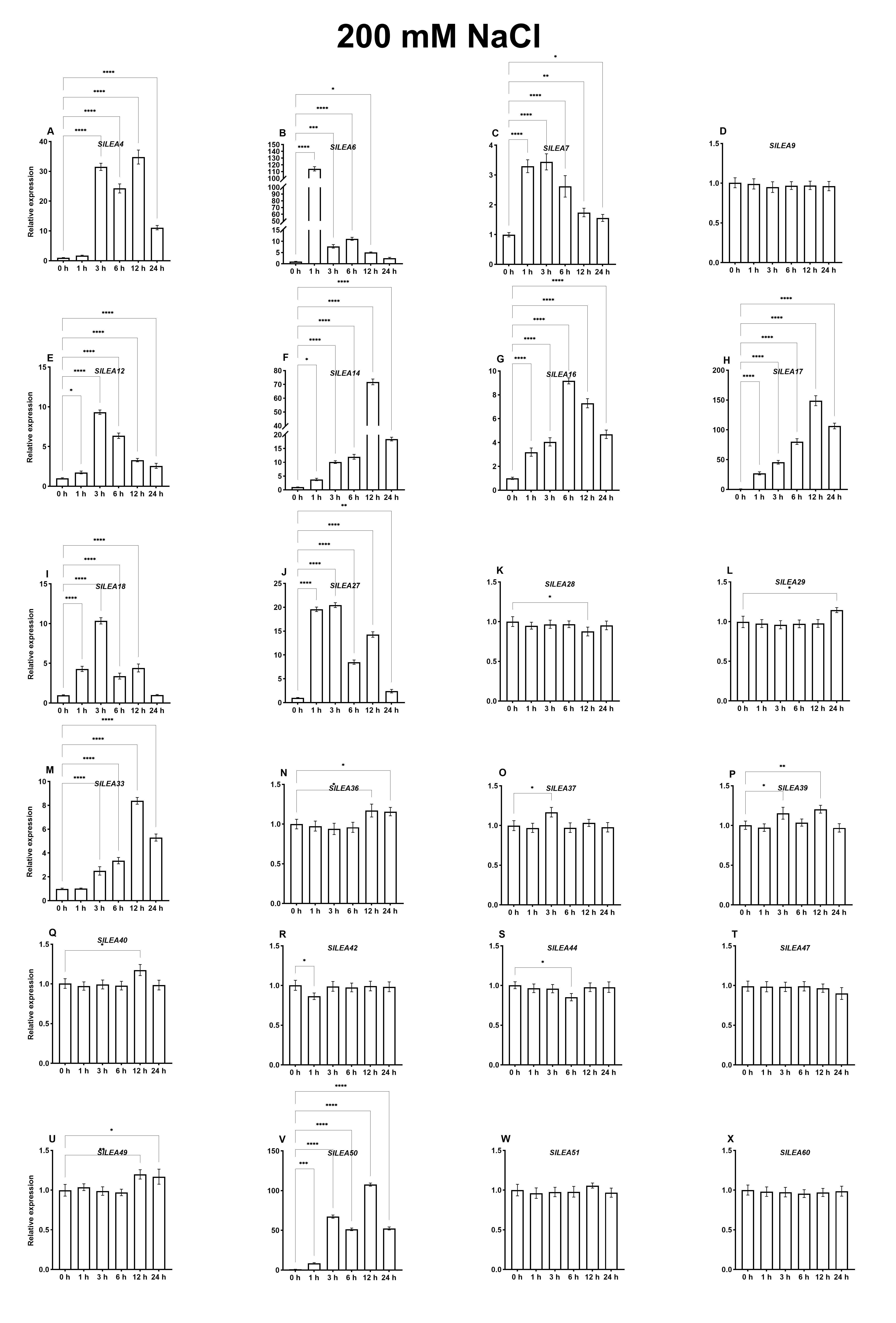

Supplement: Supplementary file 6 — Additional file 6: Figure S3. Expression patterns of SlLEAs under simulated high salt stress (200 mM NaCl). Three independent biological replicates were included to calculate the mean. Error bars show the SD of the three biological replicates. Values represent mean ± SD. Statistical significance of the differences was confirmed using Dunnett's multiple comparisons test (*P<0.05, **P<0.01, ***P<0.001, and ****P<0.0001). [file 12870_2022_3953_MOESM6_ESM.tif]

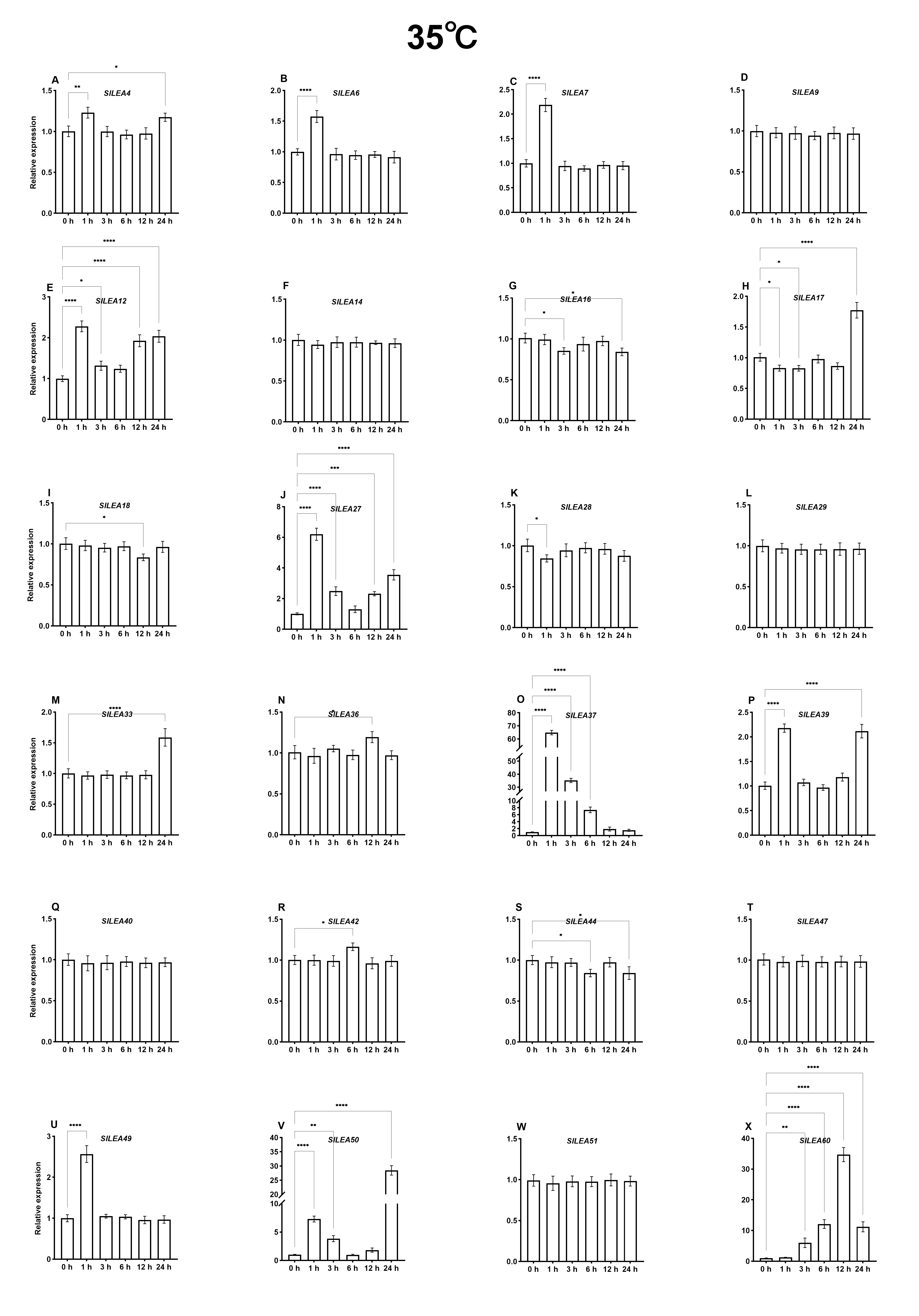

Supplement: Supplementary file 7 — Additional file 7: Figure S4. Expression patterns of SlLEAs under simulated high-temperature stress (35°C). Three independent biological replicates were included to calculate the mean. Error bars show the SD of the three biological replicates. Values represent mean ± SD. Statistical significance of the differences was confirmed using Dunnett's multiple comparisons test (*P<0.05, **P<0.01, ***P<0.001, and ****P<0.0001). [file 12870_2022_3953_MOESM7_ESM.tif]

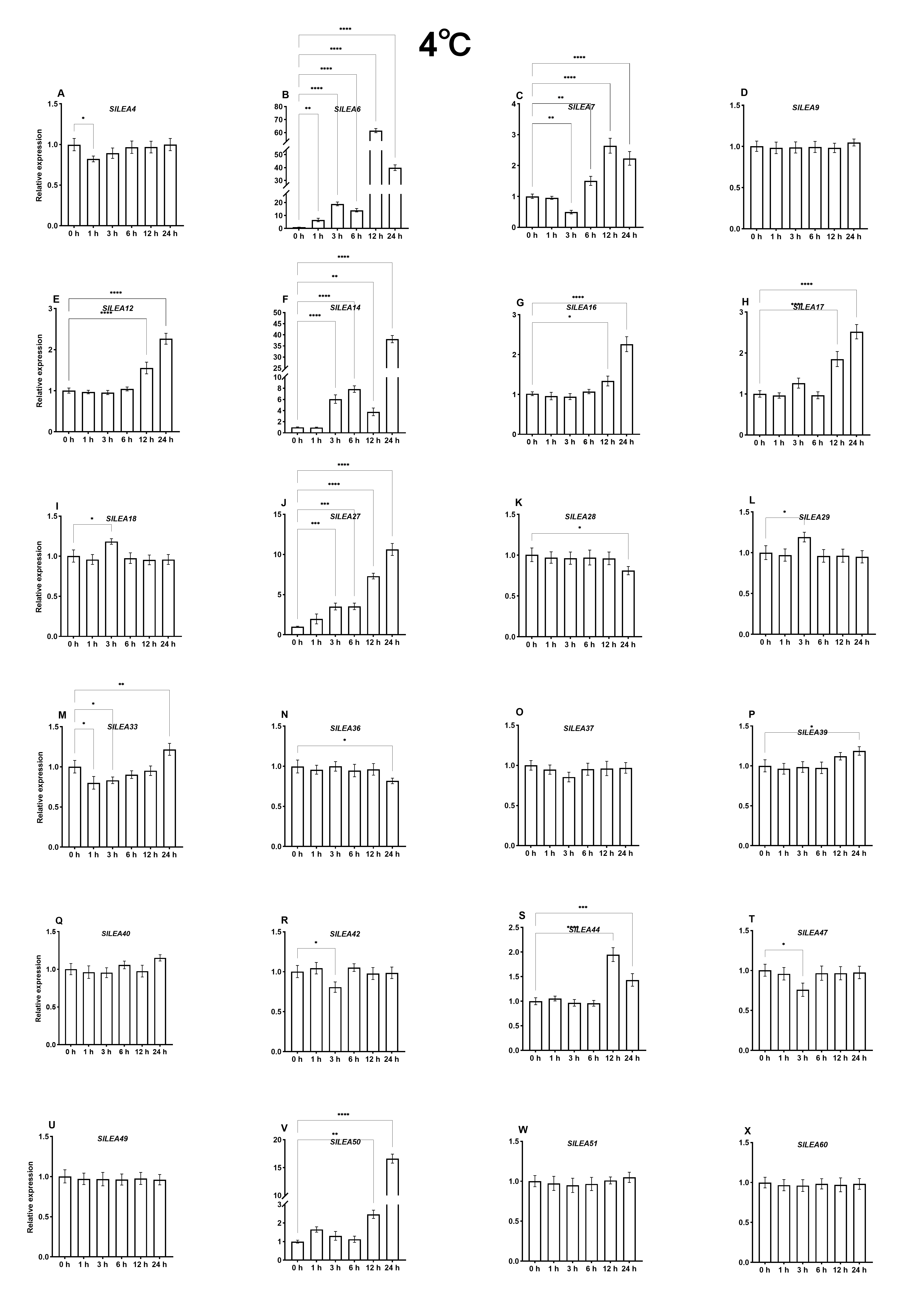

Supplement: Supplementary file 8 — Additional file 8: Figure S5. Expression patterns of SlLEAs under simulated low-temperature stress (4°C). Three independent biological replicates were included to calculate the mean. Error bars show the SD of the three biological replicates. Values represent mean ± SD. Statistical significance of the differences was confirmed using Dunnett's multiple comparisons test (*P<0.05, **P<0.01, ***P<0.001, and ****P<0.0001). [file 12870_2022_3953_MOESM8_ESM.tif]

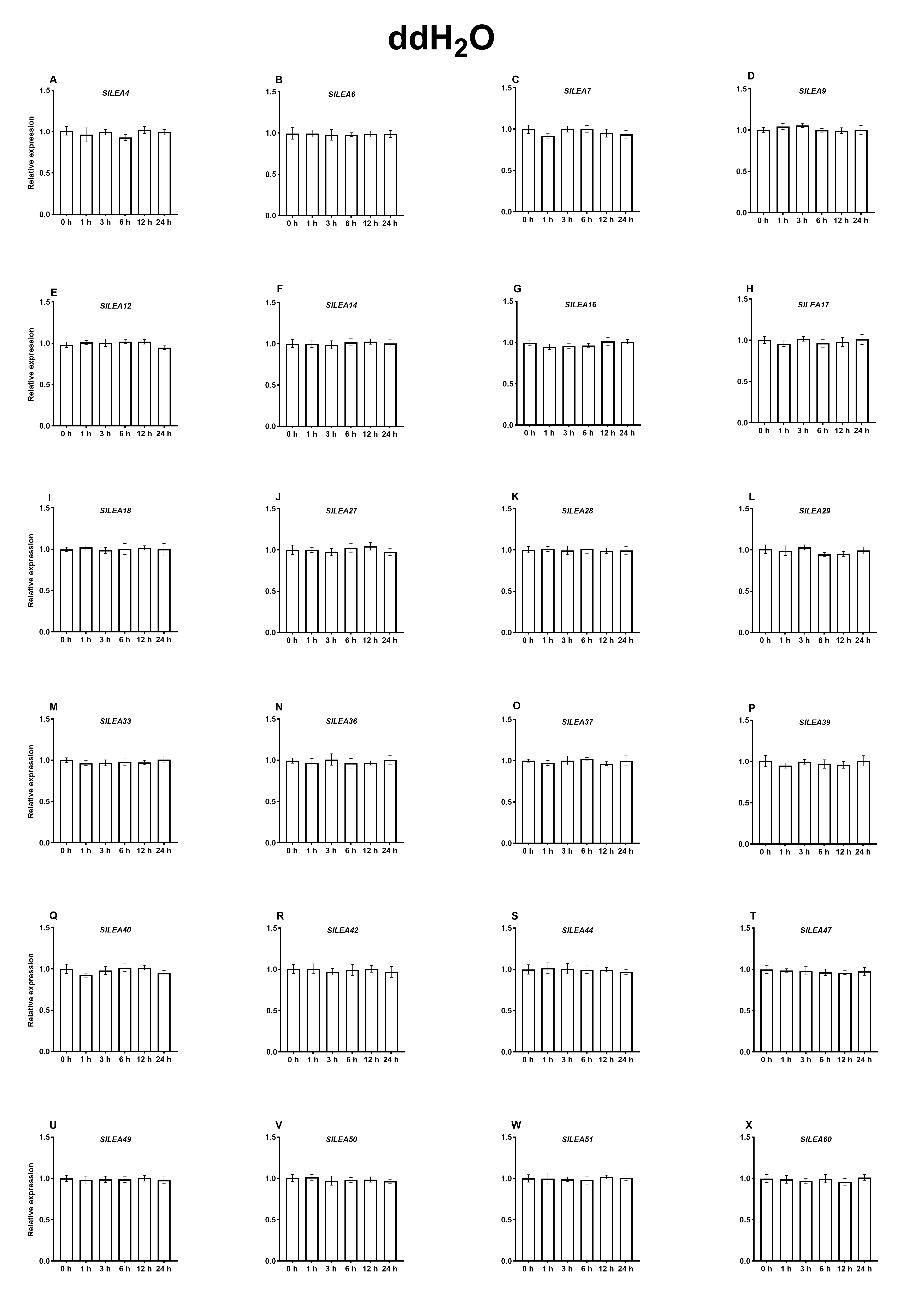

Supplement: Supplementary file 9 — Additional file 9: Figure S6. Expression patterns of SlLEAs in response to ddH2O control treatment. Three independent biological replicates were included to calculate the mean. Error bars show the SD of the three biological replicates. Values represent mean ± SD. Statistical significance of the differences was confirmed using Dunnett's multiple comparisons test (*P<0.05, **P<0.01, ***P<0.001, and ****P<0.0001). [file 12870_2022_3953_MOESM9_ESM.tif]

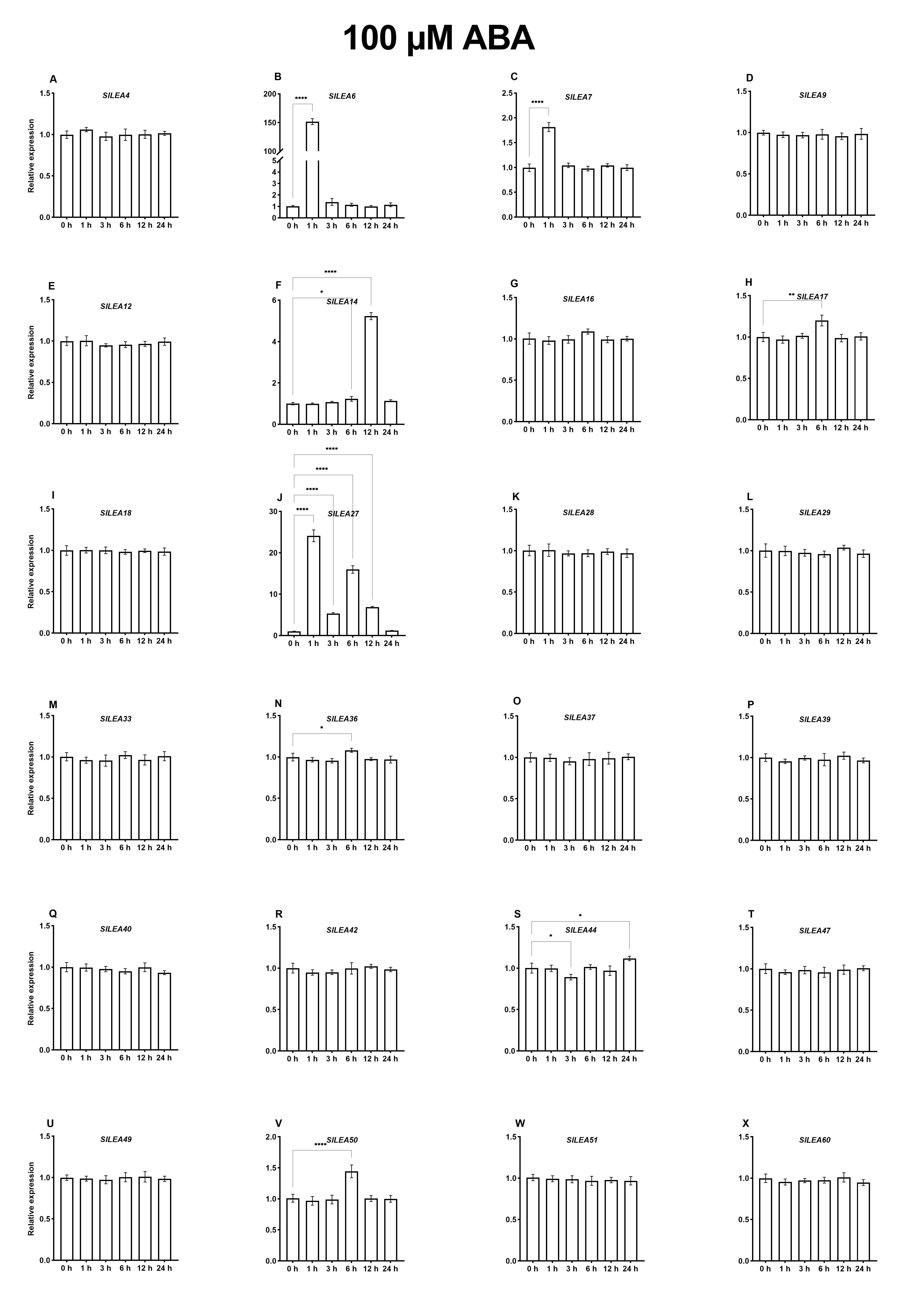

Supplement: Supplementary file 10 — Additional file 10: Figure S7. Expression patterns of SlLEAs in response to 100 µM ABA treatment. Three independent biological replicates were included to calculate the mean. Error bars show the SD of the three biological replicates. Values represent mean ± SD. Statistical significance of the differences was confirmed using Dunnett's multiple comparisons test (*P<0.05, **P<0.01, ***P<0.001, and ****P<0.0001). [file 12870_2022_3953_MOESM10_ESM.tif]

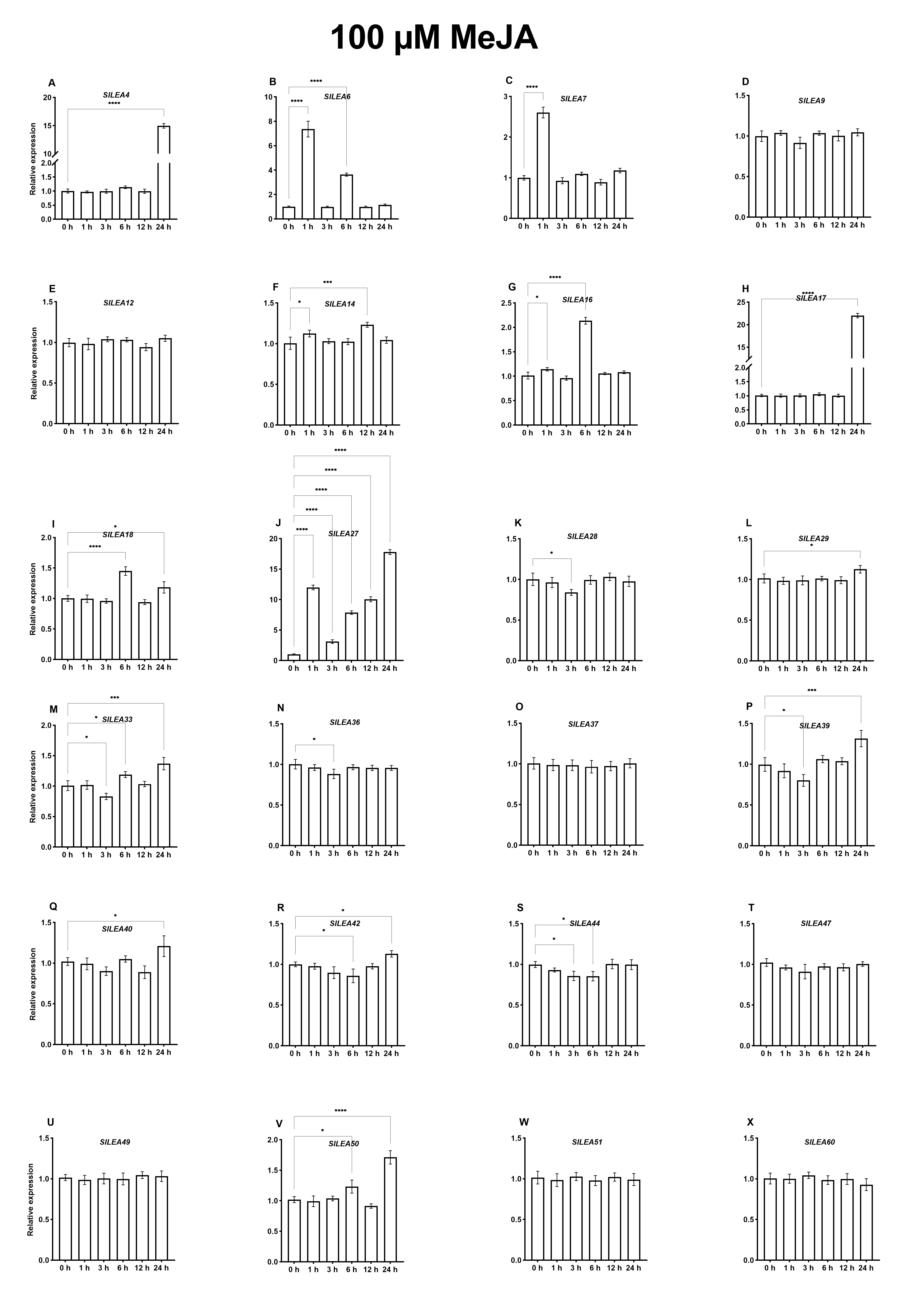

Supplement: Supplementary file 11 — Additional file 11: Figure S8. Expression patterns of SlLEAs in response to 100 µM MeJA treatment. Three independent biological replicates were included to calculate the mean. Error bars show the SD of the three biological replicates. Values represent mean ± SD. Statistical significance of the differences was confirmed using Dunnett's multiple comparisons test (*P<0.05, **P<0.01, ***P<0.001, and ****P<0.0001). [file 12870_2022_3953_MOESM11_ESM.tif]

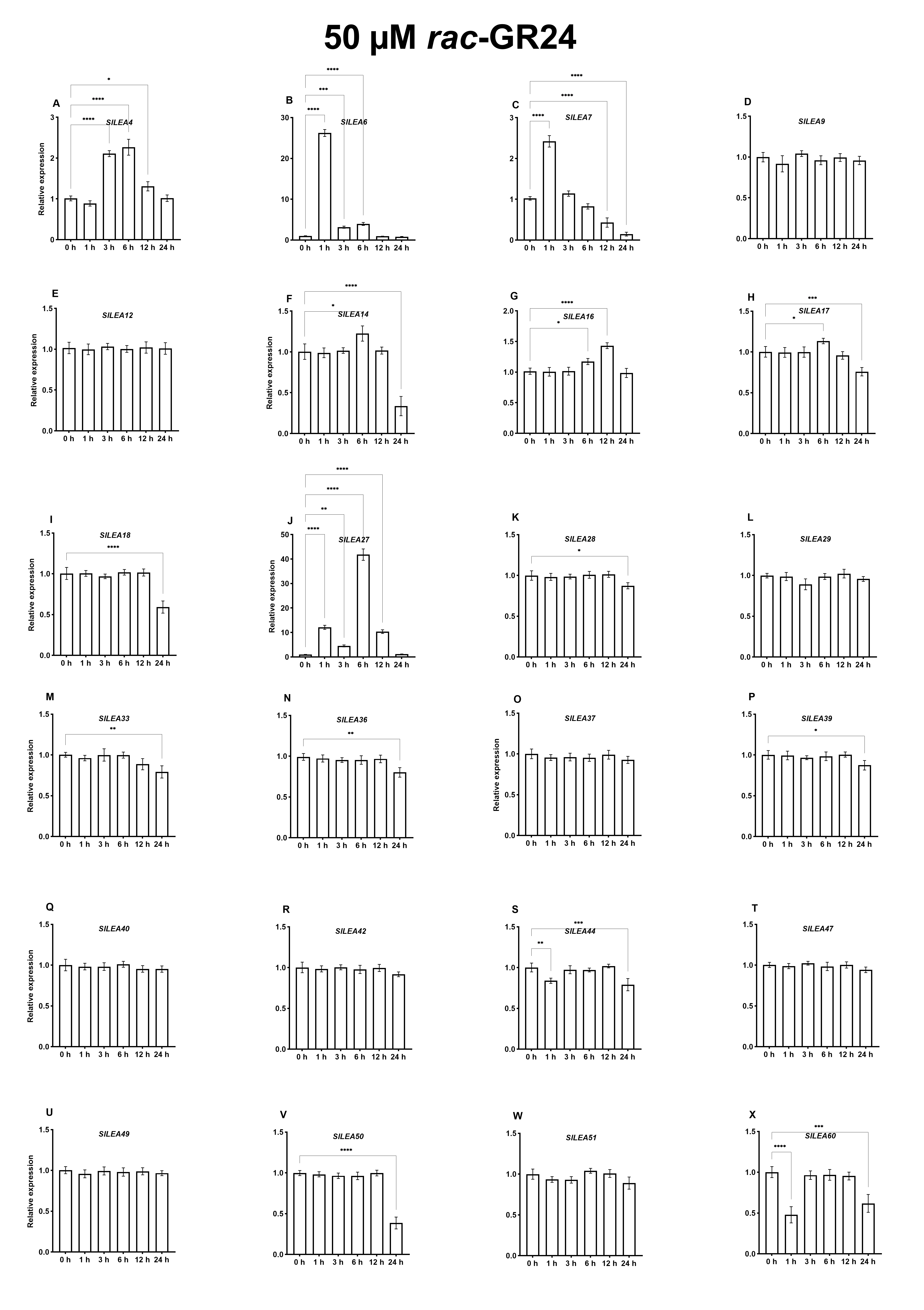

Supplement: Supplementary file 12 — Additional file 12: Figure S9. Expression patterns of SlLEAs in response to 50 µM rac-GR24 treatment. Three independent biological replicates were included to calculate the mean. Error bars show the SD of the three biological replicates. Values represent mean ± SD. Statistical significance of the differences was confirmed using Dunnett's multiple comparisons test (*P<0.05, **P<0.01, ***P<0.001, and ****P<0.0001). [file 12870_2022_3953_MOESM12_ESM.tif]

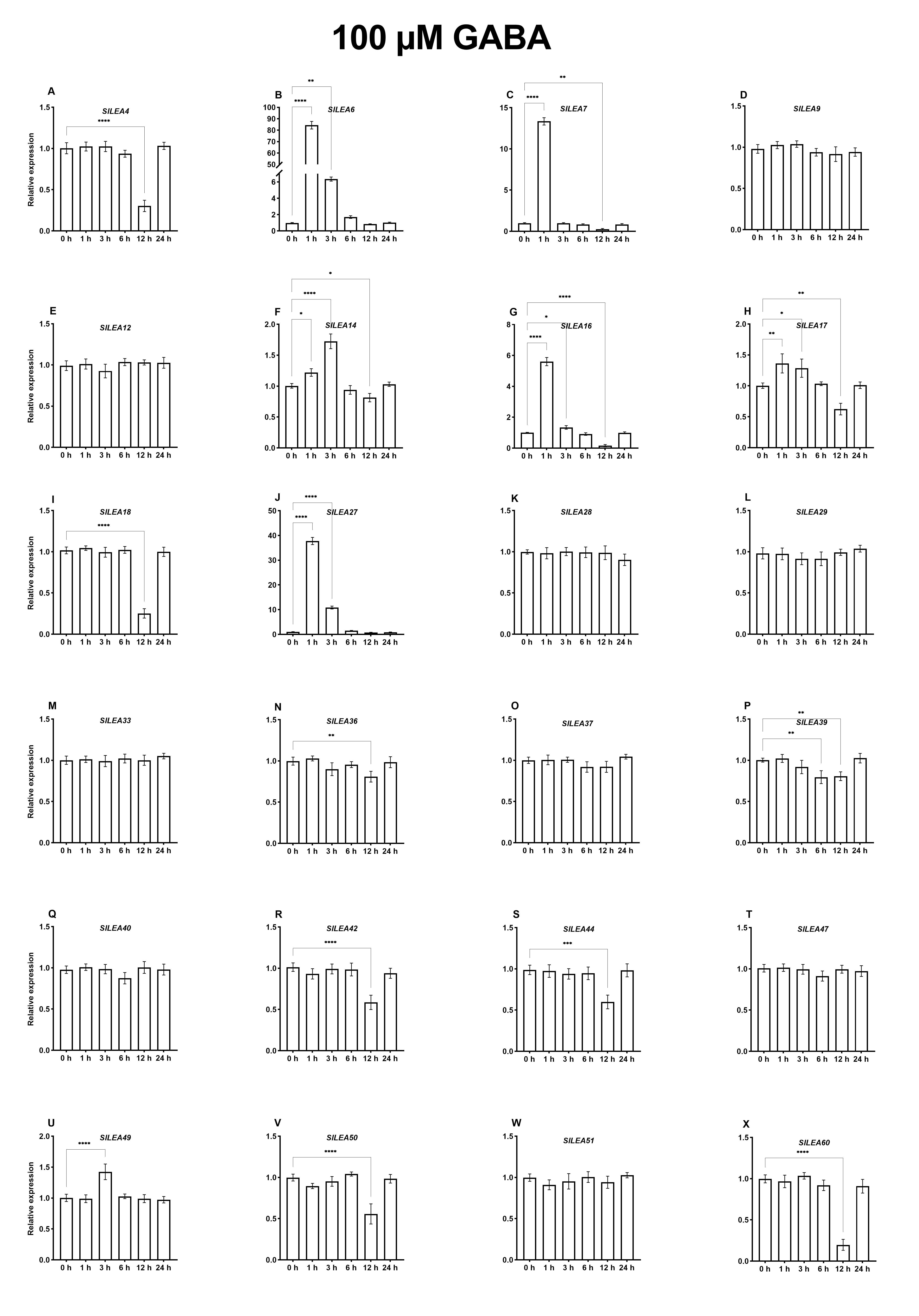

Supplement: Supplementary file 13 — Additional file 13: Figure S10. Expression patterns of SlLEAs in response to 100 µM GABA treatment. Three independent biological replicates were included to calculate the mean. Error bars show the SD of the three biological replicates. Values represent mean ± SD. Statistical significance of the differences was confirmed using Dunnett's multiple comparisons test (*P<0.05, **P<0.01, ***P<0.001, and ****P<0.0001). [file 12870_2022_3953_MOESM13_ESM.tif]

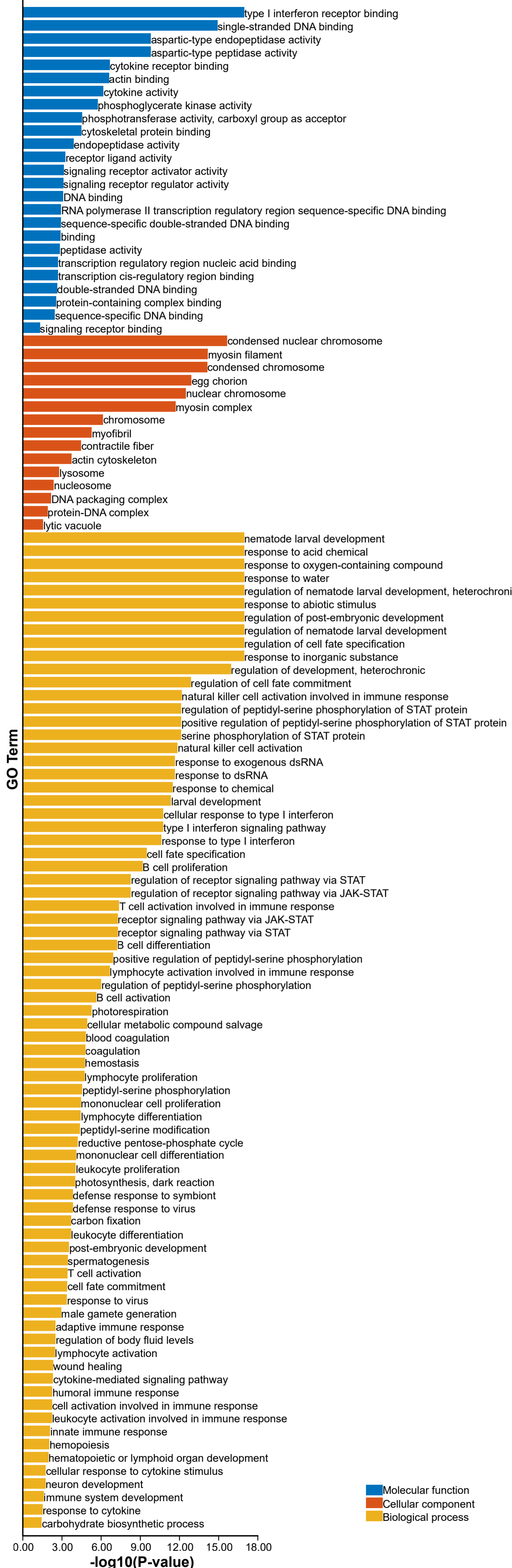

Supplement: Supplementary file 14 — Additional file 14: Figure S11. GO analysis of 254 LEAs in this study. [file 12870_2022_3953_MOESM14_ESM.pdf]

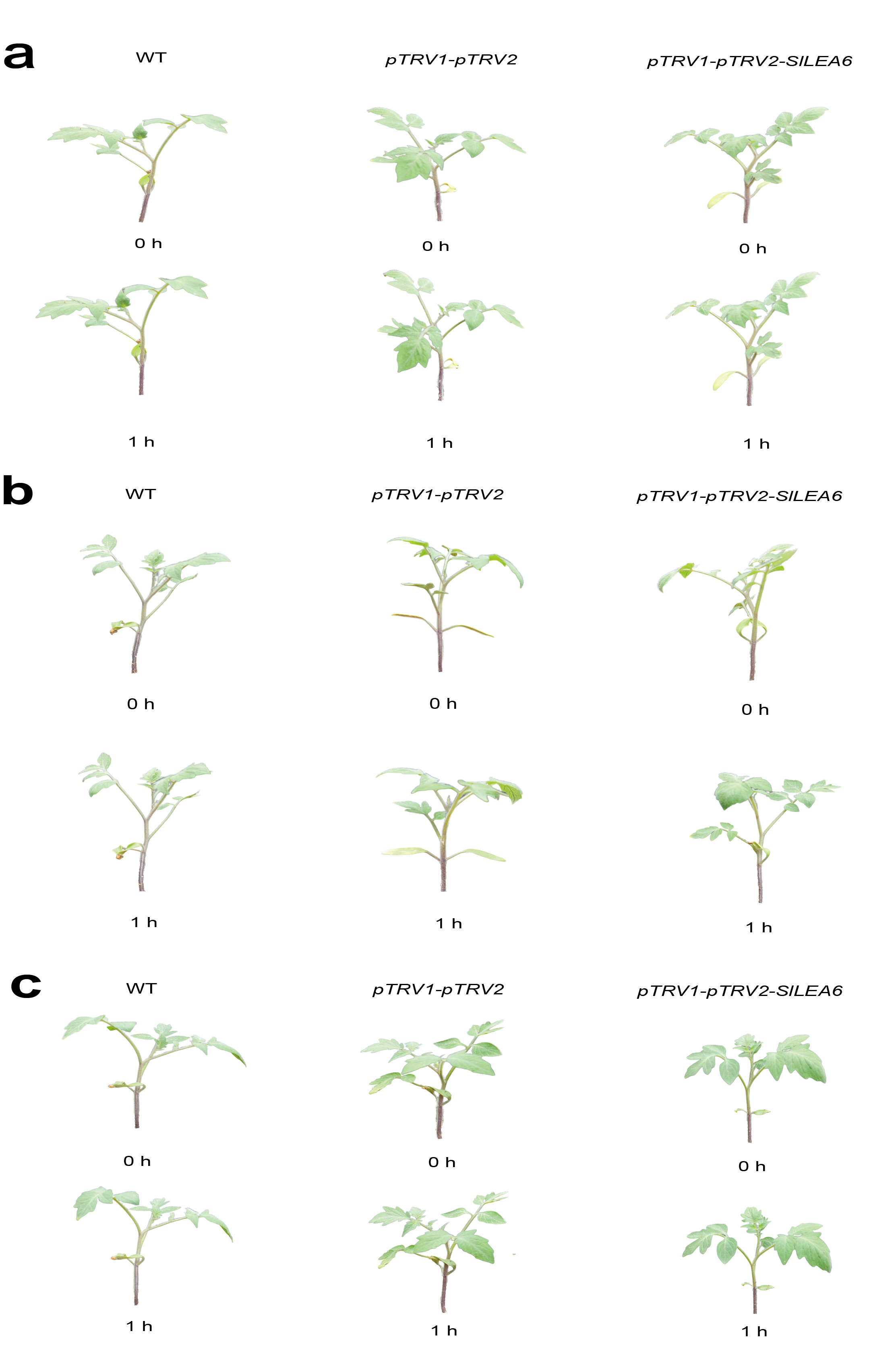

Supplement: Supplementary file 15 — Additional file 15: Figure S12. Phenotypic analysis under abiotic stress conditions after silencing of SlLEA6 (a: salt stress; b: high temperature stress; c: low temperature stress). [file 12870_2022_3953_MOESM15_ESM.tif]

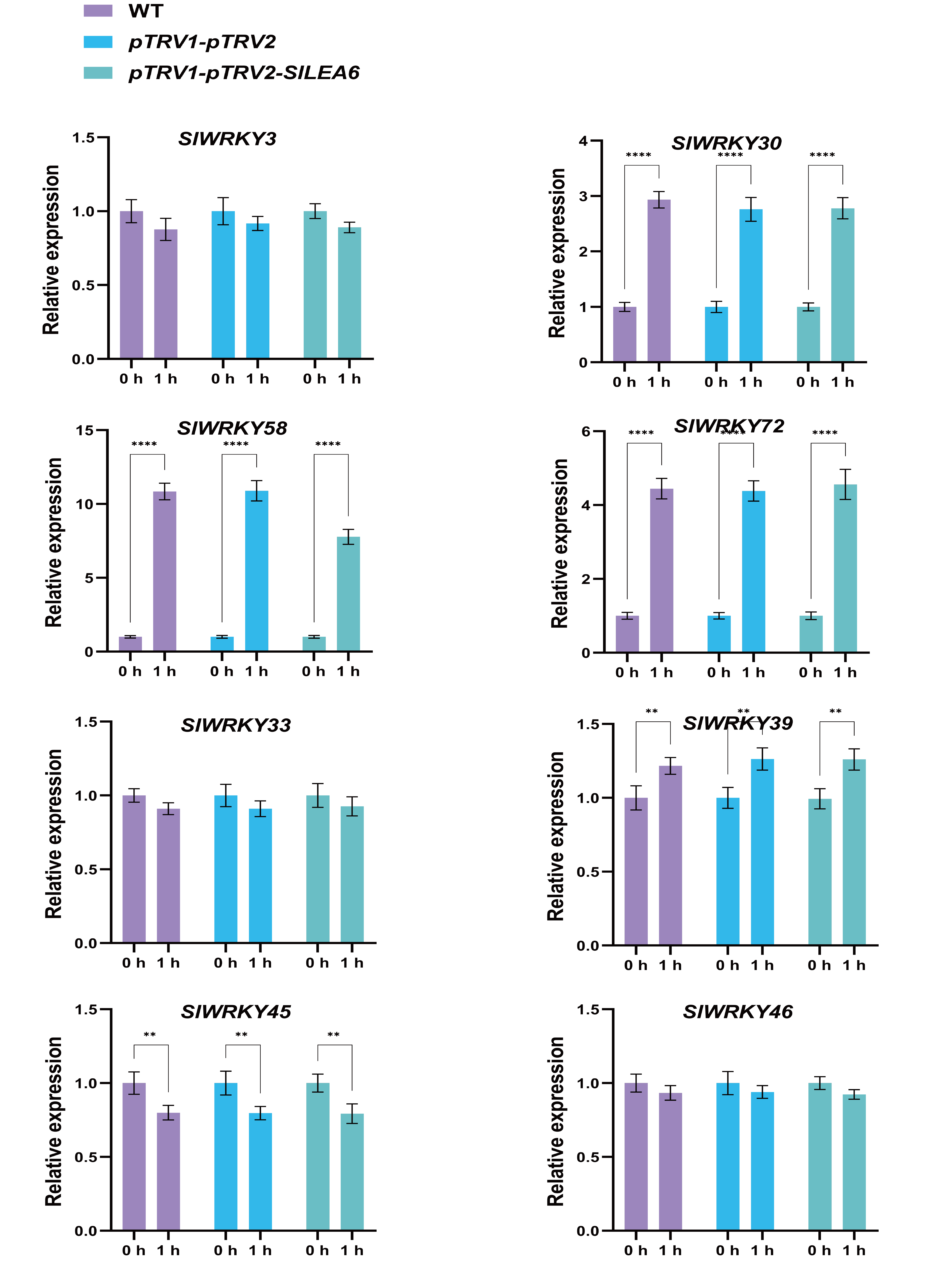

Supplement: Supplementary file 16 — Additional file 16: Figure S13. Expression patterns of eight drought-induced SlWRKYs in SlLEA6 silenced lines under drought stress. Three independent biological replicates were included to calculate the mean. Error bars show the SD of the three biological replicates. Values represent mean ± SD. Statistical significance of the differences was confirmed using Dunnett's multiple comparisons test (*P<0.05, **P<0.01, ***P<0.001, and ****P<0.0001). [file 12870_2022_3953_MOESM16_ESM.tif]
